# The interplay between type 2 diabetes mellitus and sepsis: a scoping review

**DOI:** 10.7150/ijms.126520

**Published:** 2026-06-04

**Authors:** Sarah Sharul Sham, Sok Kuan Wong, Kok-Yong Chin, Nur Liyana Mohammed Yusof, Toh Leong Tan, Evelyn Yi Wen Chau, Aireen Zamhot, Amirudin Sanip, Amir Muharram

**Affiliations:** 1 Department of Pharmacology, Faculty of Medicine, Universiti Kebangsaan Malaysia, Jalan Yaacob Latif, Bandar Tun Razak, 56000 Cheras, Kuala Lumpur, Malaysia.; 2 Department of Emergency Medicine, Faculty of Medicine, Universiti Kebangsaan Malaysia, Jalan Yaacob Latif, Bandar Tun Razak, 56000 Cheras, Kuala Lumpur, Malaysia.

**Keywords:** insulin, glucose, inflammation, immunomodulation, organ dysfunction

## Abstract

Type 2 diabetes mellitus (T2DM) influence sepsis onset and progression. This scoping review mapped 33 studies from PubMed and Scopus up to June 2025 that examined immune, genetic, metabolic, prognostic, clinical, or treatment-related outcomes in patients with T2DM and sepsis. Sepsis patients with T2DM were prone to infections. Immune dysregulation in diabetic sepsis involved sustained pro-inflammatory cytokine release, impaired anti-inflammatory responses and altered immune cell profiles. Multi-organ dysfunction was more severe in T2DM. Monocyte chemoattractant protein-1 polymorphisms increased sepsis susceptibility and amplified inflammation. Poor glycaemic control, advanced diabetes, and comorbidities worsened sepsis outcomes and prolonged hospitalisation. Mortality findings in diabetic sepsis were inconsistent. Insulin influenced sepsis outcomes by C-peptide modulation. Metformin, sodium-glucose co-transporter-2 inhibitors, dipeptidyl peptidase-4 inhibitors, and statins exhibited immunomodulatory effects. Granulocyte-macrophage colony-stimulating factor and ulinastatin restored immune function and suppressed inflammation. Larger multi-ethnic studies are warranted to define optimal glycaemic targets, uncover immune-metabolic interactions, identify biomarkers, and optimise T2DM management in sepsis.

## 1. Introduction

Sepsis is defined as a critical and potentially fatal medical emergency resulting from dysregulated host response to infection, which can lead to organ dysfunction if not promptly detected and treated 1. Sepsis causes an estimated 11 million deaths annually, accounting for 20% of global mortality 2. Although anyone can be affected, certain risk factors increase susceptibility. These factors include diabetes, immunosuppression, prolonged hospitalisation, malignancy, advanced age, and haemodialysis 3. Given the crucial role of host immune response in sepsis development, immune heterogeneity among patients adds substantial complexity to its pathophysiology. In addition to the innate and adaptive immune responses, endothelial activation and coagulation pathway also contribute to inflammation and the onset of sepsis 4.

Type 2 diabetes mellitus (T2DM) is a chronic disease characterised by hyperglycaemia resulting from insulin resistance 5. Its prevalence is rising, reaching 10.8% in the Southeast Asia region 6. The hyperglycaemic internal milieu fosters an environment ideal for pathogen growth 7. If left unaddressed, it can lead to oxidative stress-induced tissue damage 8. Persistently elevated blood glucose level in T2DM impairs normal immune and physiological functions through various mechanisms. These include downregulated neutrophil migration and phagocytic activity, increased neutrophil apoptosis, vasodilation and oedema due to increased blood viscosity, hypoxia resulting from platelet aggregation, and metabolic acidosis caused by impaired glucose metabolism. These dysfunctions collectively heighten infection susceptibility 8, 9. Hyperglycaemia also reprograms neutrophils and macrophages by altering their metabolic activity. This phenomenon is associated with 'trained immunity', in which innate immune cells adopt sustained and exaggerated inflammatory response resembling immune memory 10. The persistent inflammatory state in T2DM further exacerbates adipose tissue inflammation and pancreatic ꞵ-cells dysfunction, contributing to disease progression 11. Clinically, these pathophysiological alterations may manifest as more pronounced organ dysfunction which is captured by elevated clinical severity scores, such as sequential organ failure assessment (SOFA) and acute physiology and chronic health evaluation II (APACHE II) in diabetic sepsis patients compared to non-diabetic sepsis patients.

Several meta-analyses have examined the impact of T2DM on sepsis outcomes but yielded conflicting findings. One study reported that sepsis patients with diabetes had lower mortality and shorter hospital stays than non-diabetics, despite a higher incidence of acute kidney injury (AKI) 12. Conversely, a more recent systematic review and meta-analysis of 21 studies reported no significant differences in in-hospital mortality, 90-day mortality, respiratory failure, cardiac events, readmissions, and length of stay between diabetic and non-diabetic patients. Nonetheless, the risk of acute renal failure remained higher among diabetic individuals 13. Additionally, a Mendelian randomisation study by Wang et al. challenged the commonly held belief that T2DM increased infection risk, showing no causal relationship between T2DM and susceptibility to infection 14. While the metabolic and immune alterations in T2DM have been extensively explored, its role in sepsis pathogenesis remains unclear. Given the growing prevalence of T2DM, the fatality of sepsis, and inconclusive findings from previous research, there is a pressing need to clarify their relationship. Therefore, a scoping review is necessary to map existing evidence and identify knowledge gaps on the relationship between T2DM and sepsis for future research directions.

This review summarises existing evidence on the relationship between T2DM and sepsis, with an emphasis on how T2DM influences key biological mechanisms and clinical outcomes. It also explores the impact of commonly prescribed antidiabetic medications, lipid-modulating therapies, and other relevant treatments on sepsis progression. Specifically, this scoping review aims to address the following research questions: (1) “What is the current evidence on the relationship between T2DM and sepsis?”; (2) “How does T2DM influence infection susceptibility, inflammatory responses, oxidative stress, immune function, pathological changes, genetic factors, prognostic and clinical outcomes in sepsis patients?”; (3) “How do antidiabetic and related therapeutic interventions affect the development and progression of sepsis?”. The findings of this review are expected to guide future clinical research and support the development of early detection and intervention strategies for sepsis among patients with T2DM.

## 2. Methodology

This scoping review was conducted in accordance with the Preferred Reporting Items for Systematic Reviews and Meta-Analyses Extension for Scoping Reviews (PRISMA-ScR) guidelines to ensure methodological rigor and transparency (Table S1). The review was conducted through a structured five-stage process, including (a) development of research question, (b) systematic retrieval of pertinent literature, (c) rigorous screening and eligibility assessment, (d) structured data extraction, and (e) comprehensive synthesis and presentation of the findings. The protocol of this scoping review has been registered in the Open Science Framework (Url: https://osf.io/yag29/overview?view_only=182ee718fdbf47478fc362014f577e7).

### 2.1 Literature search

The search strategy was developed using a combination of relevant keywords, synonyms and combined vocabulary combined with Boolean operators. The core concepts included “type 2 diabetes mellitus” and “sepsis”, along with related terms such as “T2DM”, “septicaemia” and “septic shock”. A comprehensive literature search was conducted on 1^st^ June 2025 using PubMed and Scopus databases to retrieve articles published from 2000 up to 2025. These databases were selected due to their extensive coverage of biomedical and interdisciplinary literature. They are also commonly used in structured reviews. PubMed provides access to MEDLINE-indexed studies with controlled vocabulary indexing to enhance sensitivity for medical topics. On the other hand, Scopus offers broad multidisciplinary coverage and citation tracking to improve retrieval of studies not indexed in PubMed. Together, these two databases capture a substantial proportion of relevant peer-reviewed studies. The detailed search strategies for each database are provided in Supplementary Table S2.

### 2.2 Eligibility criteria

The research question guiding this review was: “What is the relationship between T2DM and sepsis in terms of infection susceptibility, inflammatory and oxidative stress responses, immune function, genetic influences, clinical and prognostic outcomes, as well as effects of relevant therapeutic interventions on sepsis-related outcomes?”.

Studies were selected based on the population, concept and context framework. The population referred to patients with T2DM who developed sepsis. The concept focused on five main domains: (a) susceptibility to infection, (b) inflammatory, oxidative stress and immune mechanisms, (c) genetic influences, (d) clinical and prognostic outcomes, and (e) therapeutic interventions. The context encompassed both preclinical and clinical investigations, allowing comprehensive mapping of evidence across the translational spectrum and facilitating identification of gaps between mechanistic and clinical research.

Studies were considered eligible if they met the following criteria. First, the study population consisted of patients with T2DM who were diagnosed with sepsis or relevant experimental models of diabetic sepsis. Second, the study examined at least one outcome related to immune responses, genetic and metabolic factors, prognostic markers, pathological or clinical outcomes of sepsis, as well as the effects of therapeutic interventions in context of diabetic sepsis. Studies focusing exclusively on sepsis progression or management in non-diabetic populations were excluded. Third, studies were conducted in clinical, hospital or laboratory settings, where diabetic sepsis was explicitly diagnosed and assessed. Animal studies were also included to provide mechanistic and pathophysiological insights relevant to diabetic sepsis. Finally, only peer-reviewed articles published in English were included to ensure accurate interpretation of methodology and outcome reporting.

Grey literature was excluded in the search strategy as the primary objective of this review was to synthesise evidence from peer-reviewed primary studies to ensure methodological rigor, data reliability, and consistency of reporting. Given the broad scope and mechanistic emphasis of the research questions, this review prioritised studies with detailed experimental and clinical data which are typically available in indexed journals.

### 2.3 Study selection

All retrieved articles were exported into EndNote 2025 (Clarivate) for reference management and deduplication. Title/abstract screening and full-text assessment were conducted independently by two reviewers (S.S.S. and S.K.W.). Data extraction was performed by one reviewer (S.S.S.) and subsequently validated by a second reviewer (S.K.W.) to ensure accuracy and completeness. Any discrepancies during screening or data extraction were resolved through discussion and consensus, with involvement of a third reviewer (T.L.T.) when necessary.

As this scoping review aimed to map existing body of evidence rather than to estimate pooled effects, multiple publications originating from the same registry or cohort were included. Potential overlap between study populations was acknowledged and publications were not treated as independent sources of evidence. Data extraction and synthesis were therefore conducted at the level of individual studies to reflect patterns of research activity, study characteristics, and reported outcomes, rather than the strength of evidence based on unique patient populations.

### 2.4 Data extraction

Data extraction involved recording information in tabular format for author/year, characteristics of the animals/subjects involved, sample size, age, and key findings relevant to diabetic sepsis outcomes.

### 2.5 Risk of bias assessment

As this study was conducted as a scoping review, no formal risk-of-bias or quality appraisal was undertaken. This approach is consistent with the methodological guidance for scoping reviews, which primarily aim to map the existing evidence, identify key concepts, and explore knowledge gaps rather than critically appraise study quality.

### 2.6 Synthesis of results

A narrative synthesis was performed to summarise the findings related to immune modulation, inflammation pathways, metabolic, genetic and prognostic markers, pathological and clinical outcomes, and impacts of relevant treatments on diabetic sepsis outcomes. Studies were grouped according to study design, including *in vivo* and human studies. Studies involving human participants were further categorised based on their respective study design.

## 3. Results and Discussion

### 3.1 Study selection

**Figure 1:**
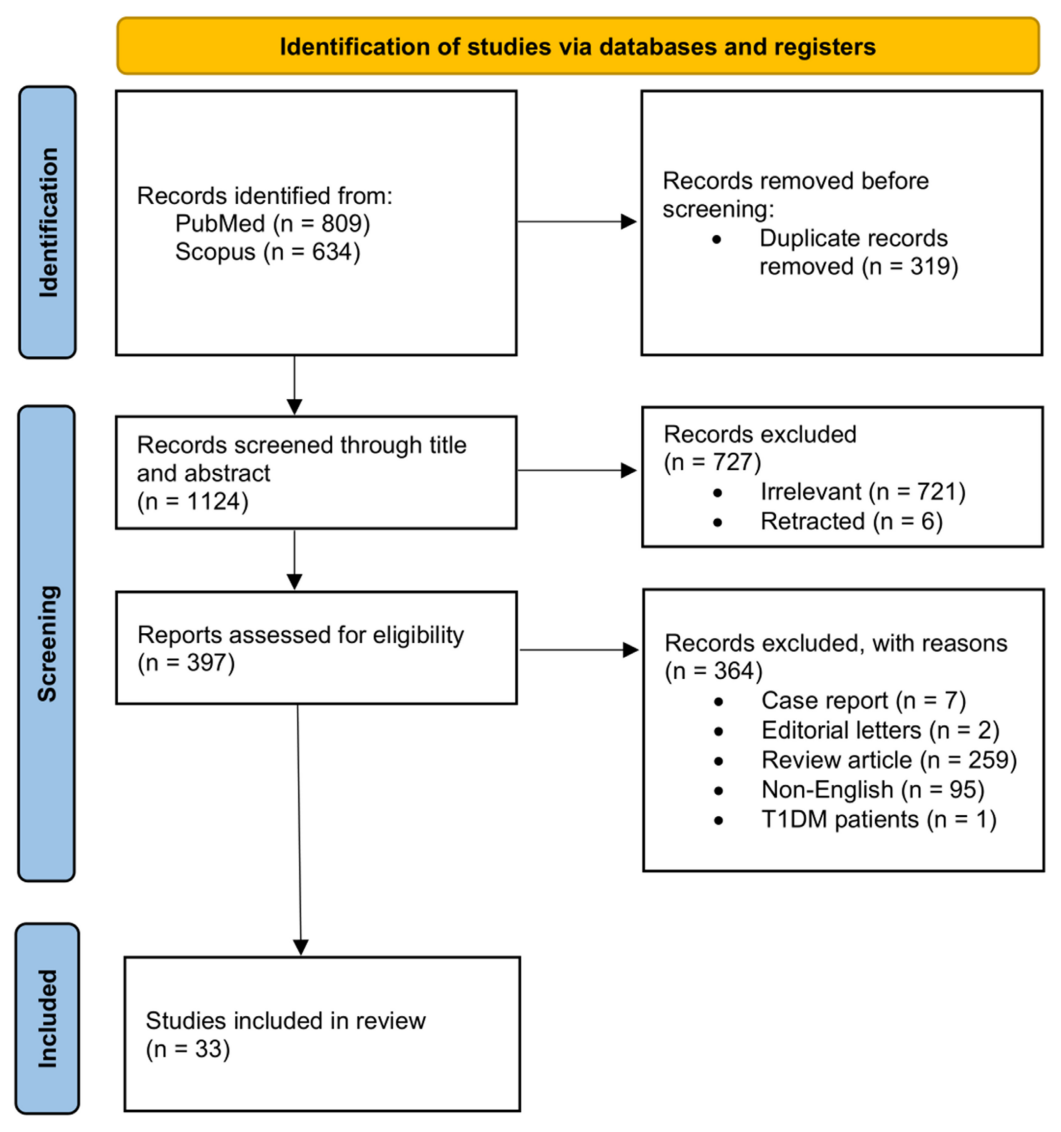
PRISMA flowchart depicting the literature search and study selection process for the scoping review, including the initial search of PubMed and Scopus databases conducted on 1^st^ June 2025 followed by title and abstract screening full-text review, and final study inclusion.

**Figure 1** illustrates the PRISMA flowchart depicting the overall study selection process. The initial search yielded 1,443 studies (PubMed = 809; Scopus = 634), of which 319 were removed after deduplication. The remaining 1,124 studies underwent title and abstract screening, resulting in the exclusion of 727 studies for not meeting the predefined criteria. The remaining 397 studies were then assessed for eligibility through full-text screening. Of those, 33 studies were included whereas 364 studies were excluded for specific reasons, including case reports (n=7), editorial letters (n=2), review articles (n=259), non-English publications (n=95) and wrong population (n=1).

### 3.2 Study characteristics

Tables 1, 2, 3 and 4 summarise the characteristics and outcomes of all 33 included studies, organised according to study design. Four studies utilised *in vivo* animal models 15-18 (Table 1), nine were prospective observational studies 19-27 (Table 3), and nineteen were retrospective observational studies 28-46 (Table 4). Additionally, one study reported the outcomes derived from both an *in vivo* animal model and a bioinformatics case-control analysis of publicly available microarray datasets 47 (Table 1). The publication years of the included studies ranged from 2007 to 2025. The focus of these included studies was to elucidate the molecular mechanisms involved in diabetic sepsis, understand sepsis outcomes and mortality in T2DM, explore common microbiological and clinical features, identify relevant biomarkers and predictive models, and investigate therapeutic and drug associations with sepsis outcomes in diabetic patients. Several included studies were derived from the same registries or cohorts. Specifically, two studies originated from the same hospital cohort at Shamir Medical Centre, five studies used the National Health Insurance Research Database (NHIRD), two studies were based on the Medical Information Mart for Intensive Care IV (MIMIC-IV), and one study used the Medical Information Mart for Intensive Care III (MIMIC-III). As this review aimed to comprehensively map the literature, overlapping studies were not excluded. However, findings were interpreted at the level of individual publications rather than independent cohorts. No quantitative pooling or meta-analysis was performed.

In animal model studies (Table 1), three studies utilised male C57BL/6 mice, one study used Sprague-Dawley rats, and one study used Goto-Kakizaki rats. In C57BL/6 and Sprague-Dawley rats, T2DM was typically induced using a high-fat diet and/or a streptozotocin (STZ) injection. This induction approach led to the development of hyperglycaemia, hyperinsulinaemia, insulin resistance, and glucose intolerance in the animals 15, 16, 18. The Goto-Kakizaki rat is a well-established non-obese animal model of T2DM that develops spontaneous diabetes, characterised by high blood glucose, insulin resistance and impaired insulin secretion due to genetic defects 17, 48. On the other hand, sepsis was induced in these T2DM animal models using either caecal ligation puncture (CLP) or lipopolysaccharide (LPS) injection.

Table 2 summarises the key characteristics of the included clinical studies in this review, detailing the study period, country, study design, population, and diagnostic criteria for sepsis and T2DM. Overall, the evidence was derived from prospective and retrospective observational study designs conducted across diverse geographical locations, including Asia, Europe, Australia, the Middle East, and North America. Study populations varied substantially in size, ranging from small single-centre cohorts to large nationwide database studies involving several million participants, and generally included adult patients aged 16 years and above. Sepsis definitions were heterogeneous, encompassing established consensus criteria such as Sepsis-2 and Sepsis-3, clinician-diagnosed sepsis, and administrative coding systems including International Classification of Diseases, Ninth Revision, Clinical Modification (ICD-9-CM), Tenth Revision, Clinical Modification (ICD-10-CM) codes and International Classification of Diseases, Ninth Revision, Australian Modification (ICD-9-AM). Similarly, T2DM was defined using a range of approaches, including American Diabetes Association diagnostic criteria from different guideline years, ICD coding algorithms, prescription records, and pre-existing clinician diagnoses, with several studies not specifying formal diagnostic criteria. Two studies also did not clearly specify the type of diabetes 38, 44.

In the prospective (Table 3) and retrospective observational studies (Table 4)**,** data were collected at the hospital, population or nationwide level. Although the primary focus of this review was T2DM, two studies did not specify the type of diabetes involved 38, 44. The metabolic parameters evaluated in diabetic patients include blood glucose, glycated haemoglobin (HbA1c), insulin, diabetes complications severity index (DCSI), C-peptide, C-peptide to insulin ratio, glucagon-like peptide-1 (GLP-1), glycoprotein complement C1q tumour necrosis factor-related protein 1 (CTRP1), and cortisol levels 20, 21, 24-26, 34.

In this review, findings from preclinical and clinical studies are categorized into six key areas: **(1)** infection susceptibility, **(2)** inflammatory response, oxidative stress, and immune function, **(3)** pathological outcomes, **(4)** genetic influences on sepsis susceptibility, **(5)** prognostic and clinical outcomes, as well as **(6)** therapeutic interventions and sepsis progression in T2DM patients.

### 3.3 Infection susceptibility

The most common sites of infection in diabetic sepsis patients include the genitourinary tract, respiratory system, biliary tract, gastrointestinal tract, central nervous system, cardiovascular system, bloodstream, skin, soft tissue and bone 22, 26, 28, 29, 33, 36. Although these sites were frequently reported as sources of infection in diabetic sepsis, a study by Yang et al. reported that sepsis patients with diabetes had a higher frequency of renal, skin, soft tissue and bone infections but lower frequency of respiratory, gastrointestinal, cardiovascular, and neurological infections compared to sepsis patients without diabetes 44. The difference in infection sites between diabetic and non-diabetic sepsis patients may be attributed to various pathophysiological and clinical factors. Chronic hyperglycaemia in diabetes promotes glucosuria, creating a nutrient-rich environment that favours pathogen growth in the urinary tract 49. Furthermore, diabetic patients often require frequent hospital visits for chronic disease management, increasing their exposure to nosocomial infections, which may contribute to higher rates of skin and soft tissue infections 50. Conversely, the lower frequency of respiratory, gastrointestinal, cardiovascular, and neurological infections in diabetic patients may reflect more frequent monitoring and earlier medical interventions for complications affecting these systems. Additionally, discrepancies in infection site prevalence could be influenced by heterogeneity in study populations, including differences in sample size, geographical variation, hospital infection surveillance protocols, and diverse immune responses. The presence of dominating pathogens such as *Acinetobacter baumannii, Escherichia coli, Klebsiella spp., Pseudomonas spp., Streptococcus pneumoniae*, *Streptococcus aureus,* as well as antibiotic-resistant strains, namely extended-spectrum beta-lactamase (ESBL)-producing *Escherichia coli* and methicillin-resistant S*taphylococcus aureus* (MRSA), are commonly reported in diabetic sepsis patients 26, 31, 36. However, no significant difference was reported in the prevalence of gram-positive, gram-negative, anaerobic, atypical, or mixed infections between sepsis patients with and without T2DM 39.

### 3.4 Inflammatory response, oxidative stress, and immune function

A study by Frydrych et al. investigated the temporal dynamics of cytokine release in mice with both diabetes and sepsis. An early peak in interferon-gamma (IFN-γ) was observed as early as six hours post-CLP. At 12 hours post-CLP, interleukin-6 (IL-6) level was elevated while macrophage inflammatory protein-1 alpha (MIP-1α) level was reduced. By 18 hours post-CLP, the expressions of tumour necrosis factor-alpha (TNF-α) and interleukin-10 (IL-10) were lower in mice with diabetes and sepsis as compared to controls. For immune cell function, these mice also exhibited persistently reduced circulating neutrophils and monocytes throughout 14 days post-CLP 16. Several other *in vivo* animal studies have reported heightened systemic inflammation 20 to 24 hours after the onset of sepsis in animals with T2DM, characterised by elevated pro-inflammatory cytokines such as TNF-α, interleukin-1 beta (IL-1β), IL-6, interleukin-18 (IL-18), hypoxia-inducible factor-1 alpha (HIF-1α), myeloperoxidase (MPO), and N-acetyl-b-D-glucosaminidase (NAG) 15, 17, 18, 47. In sepsis complicated by diabetes, the inflammatory response may be driven in part by the activation of Toll-like receptor 4 (TLR4) 18. Collectively, these findings highlighted distinct temporal alterations in cytokine release followed by a dysregulated immune response in sepsis associated with T2DM. The early elevation of IFN-γ and IL-6, together with reduced IL-10 expression, suggested an amplified pro-inflammatory response coupled with impaired anti-inflammatory regulation. This imbalance could disrupt the transition from the initiation to the resolution phase of inflammation, ultimately prolonging systemic inflammation. Furthermore, the reduced levels of MIP-1α and TNF-α in the earlier stage of sepsis may suggest compromised chemotactic signalling, hindering the effective recruitment and activation of neutrophils and monocytes at infection sites. This may result in suboptimal phagocytic function and impaired bacterial clearance.

Clinical evidence has demonstrated elevated levels of inflammatory markers such as TNF-α, IL-6, and IL-1β in T2DM patients with sepsis 36. Another study by Yagmur et al. examined the role of CTRP1 and its association with metabolic and inflammatory parameters in critically ill patients admitted to the intensive care unit. CTRP1 is an adipokine that has been found to be associated with T2DM and insulin resistance 51. Among all the critically ill patients studied, those with pre-existing diabetes exhibited the highest CTRP1 level. A significant positive correlation was observed between HbA1c and CTRP1 levels. Additionally, CTRP1 was positively correlated with several inflammatory markers, including IL-6, procalcitonin, C-reactive protein (CRP), and soluble urokinase-type plasminogen activator receptor (suPAR), though no significant association was found with IL-10 27. In another study, Weng et al. identified three genes [matrix metalloproteinase-8 (MMP-8), cluster of differentiation-177 (CD177) and S100 calcium-binding protein A-12 (S100A12)] as commonly upregulated biomarkers in sepsis patients with T2DM compared to healthy controls 47. Conversely, Bloch et al. reported that sepsis patients with diabetes had lower procalcitonin levels compared to their non-diabetic counterparts 21.

Oxidative stress plays a central role in the pathophysiology of sepsis 52. It arises from an imbalance between excessive production of reactive oxygen species (ROS) and the capacity of antioxidant defence mechanisms 53. During sepsis, exaggerated inflammatory responses stimulate the overproduction of ROS, leading to cellular and tissue damage, mitochondrial dysfunction, and organ failure 54. In a preclinical model combining T2DM and sepsis induced by a high-fat diet, STZ, and LPS, the mice exhibited elevated malondialdehyde (MDA) content and reduced superoxide dismutase (SOD) activity as compared to the control mice. These findings indicated that the coexistence of hyperglycaemia and sepsis exacerbated systemic oxidative stress 18.

White blood cell count is a widely used clinical indicator for detecting infection 55. Although elevated white blood cell counts are typical in sepsis, the presence of a normal count does not preclude its diagnosis 56. No significant difference in white blood cell counts was observed between diabetic and non-diabetic sepsis patients 24. Similar findings were reported across different stages of sepsis progression 29. On the other hand, the amount of white blood cells was more abundant in the non-survivors compared to survivors 35. Lymphocytes are a subtype of white blood cells that play a central role in the adaptive immune system 57. Programmed death-1 (PD-1) is a cell surface protein expressed on T lymphocytes that functions as an immune checkpoint, delivering inhibitory signals to regulate immune responses and prevent the immune system from attacking healthy cells 58. Although the proportion of PD-1^+^ T cells did not significantly differ between sepsis patients with and without T2DM, PD-1 expression was positively correlated with sepsis severity [as assessed by APACHE II and SOFA scores] in both patient groups 24.

Furthermore, Weng et al. assessed the immune cell composition in sepsis patients with T2DM. They reported elevated proportions of neutrophils, plasma cells, monocytes, M2 macrophages, mast cells and eosinophils but reduced frequencies of CD8 T cell, memory B cells, dendritic cells, and resting natural killer cells in these patients 47. These immunological alterations may be driven by chronic hyperglycaemia and persistent low-grade inflammation in T2DM, which not only intensify immune cell recruitment and activation in response to infection but also contribute to immune cell exhaustion, apoptosis, and impaired regenerative capacity 59, 60. This dysregulation could result in an exaggerated yet functionally compromised immune response, reduced pathogen clearance, and ultimately poorer clinical outcomes in sepsis patients with diabetes.

### 3.5 Pathological outcomes

Animal studies provided valuable insights into increased vulnerability to sepsis-induced organ dysfunction, especially in the heart, kidneys, liver, and lungs. Al Zoubi et al. reported that mice on a high-fat diet and subjected to CLP exhibited marked reductions in systolic cardiac function, as demonstrated by decreased ejection fraction, fractional shortening, and functional area change. These mice also exhibited significantly elevated serum levels of creatinine, urea, alanine aminotransferase (ALT), and increased urine albumin-to-creatinine ratio. These findings indicated that pre-existing T2DM aggravated renal impairment and hepatocellular injury during sepsis 15. Pulmonary complications were also severe in diabetic septic mice. Jin et al. reported extensive lung pathology, including increased microvascular permeability, structural deformation, alveolar wall collapse, widening of the interstitium, and evidence of atelectasis, which compromises gas exchange efficiency. Histological findings revealed inflammatory cell infiltration, red blood cells within the interstitial and alveolar spaces, exfoliated bronchial epithelial cells, telangiectasia, and loss of alveolar air spaces 18. Further supporting this, Weng et al. offered further pathophysiological insights into organ damage associated with diabetes and sepsis. Histological examinations revealed lipid accumulation, inflammation, necrosis, tissue architecture disarray, and pronounced tissue deformation in the liver and kidneys. Biochemical analyses demonstrated elevated levels of lactate dehydrogenase (LDH), aspartate aminotransferase (AST), ALT, creatinine, and blood urea nitrogen (BUN) alongside reduced albumin concentration, highlighting substantial hepatic and renal damage in the sepsis with T2DM group 47.

In the clinical setting, higher grades of left ventricular diastolic dysfunction have been associated with early mortality in T2DM patients with sepsis and septic shock, suggesting underlying cardiovascular failure. Additionally, the level of cardiac biomarker N-terminal pro B-type natriuretic peptide (NT-proBNP) was significantly raised in non-survivors compared to survivors, indicating myocardial involvement 26. Diabetic sepsis patients also required higher respiratory support 33. In critically ill patients with sepsis and T2DM (where CTRP1 level was elevated and positively correlated with HbA1c), the level of CTRP1 was also associated with markers of renal function such as creatinine, urea, cystatin C, and glomerular filtration rate (GFR)-cystatin C. Besides, CTRP1 level showed correlations with bilirubin, γ-glutamyltransferase, and alkaline phosphatase, suggesting a potential link with cholestasis 27. GLP-1 is an insulinotropic hormone primarily recognised for its role in regulating blood sugar and appetite. Beyond its metabolic functions, GLP-1 also plays a crucial role in modulating the inflammatory response and has been associated with sepsis severity. Studies have shown that sepsis patients with diabetes had higher levels of both active and total GLP-1 compared to their non-diabetic counterparts 21, 25. Additionally, elevated creatinine level was observed in sepsis patients with T2DM, suggesting greater renal impairment in this group 25, 33. Li et al. compared organ function between survivors and non-survivors among sepsis patients with T2DM. The non-survivor group had higher levels of BUN, creatinine, potassium, anion gap and heart rate compared to the survival group. Consistent with these biochemical findings, both the logistic organ dysfunction system (LODS) and SOFA scores were elevated in the deceased group, reflecting more severe impairment of organ physiological functions 35. Xin et al. identified several risk factors that could predict major adverse kidney events within 30 days in sepsis patients with T2DM. Among the key predictors were mean arterial pressure, platelet count, high-density lipoprotein (HDL), apolipoprotein E (ApoE), and cystatin C levels 42. Patients with diabetes who developed sepsis were at greater risk of complications such as AKI, which was more prevalent in diabetics than non-diabetics and often necessitated acute haemodialysis 28, 39. Interestingly, although patients with both sepsis and diabetes were more likely to have renal dysfunction, they appear to have a lower relative risk of developing respiratory, haematological, and hepatic dysfunctions compared to non-diabetics 28, 44. Such observation may be attributed to several underlying mechanisms. For example, chronic hyperglycaemia, oxidative stress, and endothelial damage in diabetic conditions primarily affect the kidneys 61, potentially increasing the vulnerability to septic insult. Other organs, such as the lungs, liver, and bone marrow, may be less compromised prior to the onset of sepsis in diabetic individuals, which could explain the lower rate of dysfunction observed in these organs. Moreover, individuals with both diabetes and sepsis might exhibit impaired neutrophil functions, including reduced chemotaxis, phagocytosis, and microbicidal activity 62. These defects limit the ability of neutrophils to generate damaging oxidants 63, thereby potentially contributing to less pronounced tissue injury in specific organs.

### 3.6 Genetic influences on sepsis susceptibility

Genetic predispositions may influence immune responses, ultimately affecting the risk of sepsis development in T2DM patients. Monocyte chemoattractant protein-1 (MCP-1) is a key inflammatory mediator that facilitates the recruitment of immune cells to sites of inflammation. MCP-1 has been implicated in both T2DM and sepsis, where it contributes to insulin resistance, and serves as a potential biomarker for evaluating the severity of inflammation and organ dysfunction 64, 65. The rs1024611 AG/GG polymorphism in the promoter region of MCP-1 gene is a key genetic variant that affects its expression. Li et al. pointed out a higher prevalence of the rs1024611 AG/GG genotype in T2DM patients with sepsis compared to those without sepsis. Notably, this genotype was more frequent among patients who developed septic shock than the patients with general sepsis. The rs1024611 AG/GG polymorphism was associated with increased expression of MCP-1 and TNF-α, indicating its role in amplifying inflammatory responses and promoting sepsis progression in diabetic patients 36.

Chronic low-grade inflammation, immune dysfunction, and endothelial abnormalities are prevalent due to hyperglycaemia-induced oxidative stress and insulin resistance in T2DM. The dysregulation of MCP-1 further exacerbates these immune imbalances, creating a pro-inflammatory environment. The presence of genetic polymorphisms may amplify this effect, predisposing diabetic individuals to an exaggerated inflammatory response upon infection, leading to severe infectious complications such as sepsis. The genetic underpinnings of sepsis susceptibility in T2DM could pave the way for personalised medicine approaches. Screening for the rs1024611 polymorphism in high-risk diabetic patients may help identify individuals who are likely to develop severe inflammatory complications.

However, it remains unclear whether genetic variants alone are sufficient to trigger sepsis or whether other environmental factors (such as poor glycaemic control, obesity, or gut microbiome alterations) act synergistically to exacerbate the risk. Ethnic variations in the prevalence and impact of genetic polymorphisms warrant further investigation, as genetic susceptibility to inflammation-related diseases often differs across populations. A higher prevalence of MCP-1 rs1024611 polymorphism was noted in Koreans and Chinese individuals compared to Caucasians, highlighting ethnic disparities in sepsis and T2DM susceptibility 66. Given its role in both T2DM and sepsis, the MCP-1 rs1024611 polymorphism may serve as a promising target for genetic screening, early detection and therapeutic interventions.

### 3.7 Prognostic and clinical outcomes

The relationship between glucose-related parameters and sepsis risk has been explored. Lee et al. noticed an increased association between impaired fasting glucose and sepsis risk 45, whereas Balintescu et al. revealed a U-shape association between HbA1c level and sepsis risk in individuals with T2DM, with the lowest risk observed around an HbA1c of 53 mmol/mol. In contrast, patients with HbA1c levels below 43 mmol/mol or above 82 mmol/mol exhibited a significantly higher risk of developing sepsis 19. Elevated HbA1c level may impair immune function and increase the risk of infection, meanwhile low HbA1c level may indicate frequent hypoglycaemia or underlying frailty, both of which heighten susceptibility to sepsis 67-69. In another study, the DCSI was used to stratify the advancing disease of diabetes mellitus. They found that a higher DCSI score was associated with an increased risk of sepsis and septic shock in T2DM patients 34. These findings may suggest that the progression and severity of diabetes, rather than its mere presence, significantly contributed to sepsis risk.

T2DM patients exhibited a heavier burden of comorbidities, as indicated by higher Charlson comorbidity score 38. The common comorbidities include hypertension, stroke, heart failure, cancer, renal dialysis or transplantation, lung and liver diseases, haematological disorders, immunodeficiencies and chronic alcoholism 19, 38, 39. Among the deceased group, there was a greater prevalence of AKI, myocardial infarction, congestive heart failure, renal impairment, advanced liver disease, and metastatic solid tumours 35. The presence of these comorbidities is likely to increase the risk of infection and compromise the host's ability to mount an effective response to septic insults. Lee et al. noted patients with underlying T2DM for more than 5 years had a higher risk of developing sepsis, suggesting that hyperglycaemia-induced metabolic derangements may predispose patients to severe infections 45.

Notably, increased sepsis severity has been observed in T2DM patients as indicated by higher SOFA, APACHE II, and simplified acute physiology score II (SAPSII) values 23, 35, 38. Altered consciousness was also more frequently reported in diabetic than in non-diabetic patients 38. In addition, severe sepsis and septic shock were more frequently observed in patients with hyperglycaemia 23. These patients also experienced longer hospital stays, greater dependence on respiratory and cardiovascular support, as well as a higher need for dialysis 22, 23, 33, 35. However, another study found no significant differences in SOFA and APACHE II scores between diabetic and non-diabetic sepsis patients 24. D'Almeida et al. pointed out no correlation between random blood glucose and HbA1c levels with sepsis severity 29. Additionally, de Miguel-Yanes et al. noted that T2DM with sepsis had shorter hospital stays and were less frequently discharged home from surgical wards compared to non-diabetic patients 30. These inconsistencies may arise from heterogeneity in study design, population characteristics, and healthcare settings.

Sepsis remains a leading cause of mortality worldwide, particularly among critically ill patients. It is a life-threatening condition resulting from a dysregulated host response to infection, which progresses rapidly to organ dysfunction and death if not promptly treated 70. The influence of T2DM on sepsis-related mortality remains controversial, with studies reporting inconsistent findings. Several studies have reported no significant association between T2DM and mortality in sepsis. For instance, Balintescu et al. found that HbA1c level did not significantly correlate with mortality among individuals with T2DM who developed sepsis 19. Similarly, Jia et al. reported no difference in 28-day mortality between diabetic and non-diabetic sepsis patients 24. Despite the elevated fasting blood glucose, postprandial blood sugar, and HbA1c levels in diabetic sepsis patients, these parameters were not significantly associated with mortality 26. Sathananthan et al. also concluded that neither T2DM nor glucose level at admission was a significant predictor of mortality; instead, elevated in-hospital glucose levels were positively associated with mortality 39. In contrast, higher mortality associated to greater weight loss was observed in a well-controlled preclinical study involving male mice fed with a high-fat diet and subjected to CLP 16. In a human study, diabetic patients with sepsis who died had higher admission HbA1c levels than survivors, with HbA1c correlating with increased hospital mortality 22. Hsieh et al. observed higher odds for mortality among sepsis patients with T2DM, with hospital mortality rates increasing with increasing DCSI scores. However, similar to other studies, the initial blood glucose and HbA1c levels did not predict mortality 33. Perl et al. observed significantly higher creatinine levels in non-surviving compared to surviving sepsis patients with T2DM, suggesting that impaired renal function may contribute to mortality in this group 25. Additional factors shown to be associated with increased mortality in sepsis include advanced age, cirrhosis, prolonged intensive care unit stay, transplant history, higher SOFA and LODS scores, vasopressin use, higher mean arterial pressure, and elevated BUN level 35, 39. Interestingly, de Miguel-Yanes et al. reported a lower in-hospital mortality rate among sepsis patients with T2DM compared to their non-diabetic counterparts. They also observed a significant increase in obesity prevalence over time within the T2DM population 30. Although the exact mechanism underlying this apparent protective effect remained unclear, the authors hypothesised that chronic hyperglycaemia and obesity might confer a survival advantage. This could be attributed to elevated leptin levels in obesity, which might attenuate the endotoxin-induced inflammatory response as well as hormonal changes associated with diabetes or the effects of antidiabetic therapies 30, 71.

The inconsistent findings on the association between T2DM and sepsis-related mortality underscored the complexity and multifactorial nature of this relationship. Importantly, heterogeneity in the assessment of glycaemic parameters across studies may partly account for these discrepancies. For instance, some studies evaluated pre-sepsis glycaemic control (HbA1c) whereas others focused on acute hyperglycaemia (such as admission or in-hospital blood glucose levels). Studies assessing pre-sepsis glycaemic control generally demonstrated inconsistent or weak associations with mortality, while those examining in-hospital blood glucose levels more consistently reported positive associations with mortality. These findings suggested that acute metabolic derangements during sepsis may be more clinically relevant predictors of sepsis outcomes than chronic glycaemic status alone.

**Figure 2:**
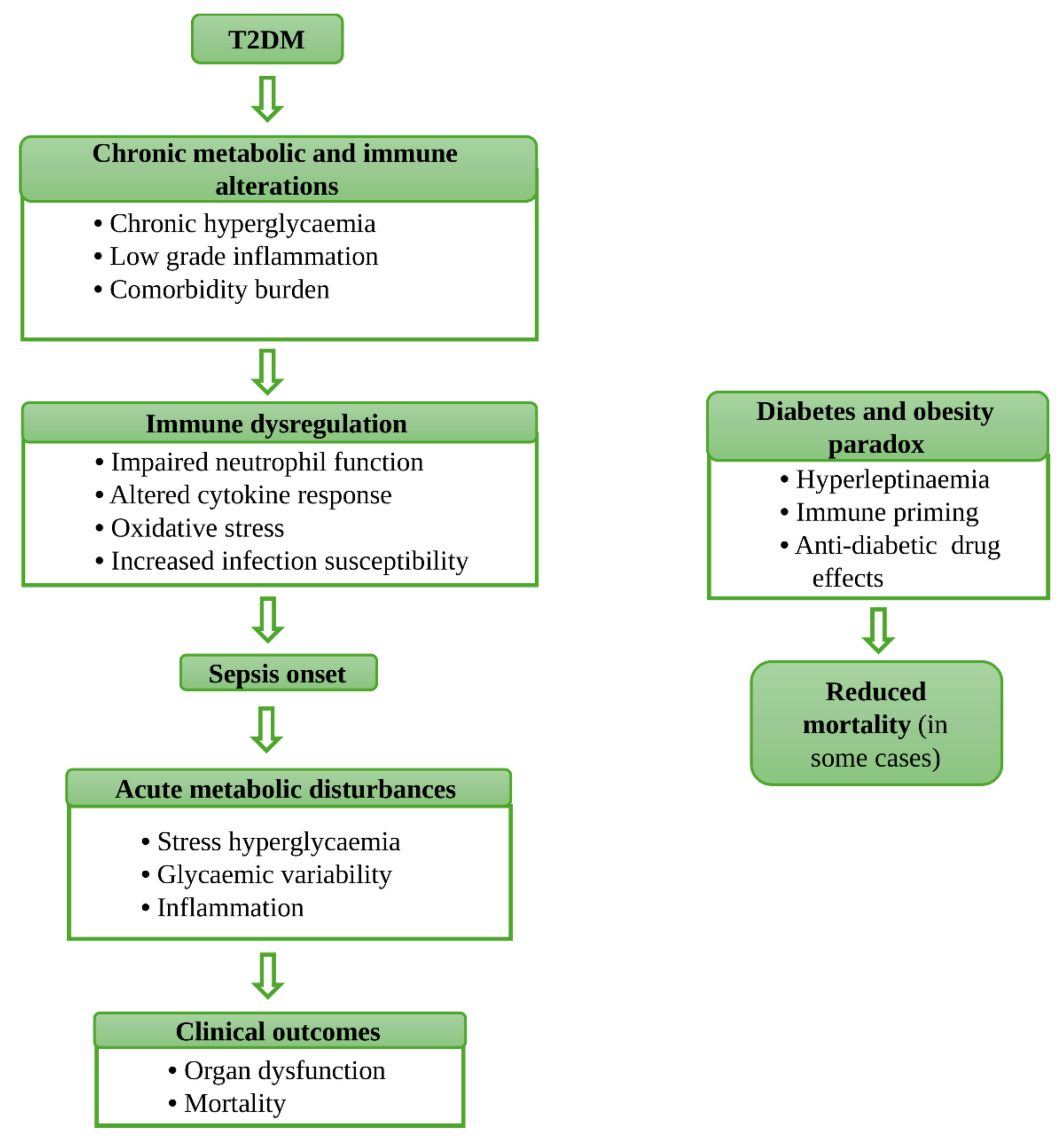
Conceptual overview of the interplay between T2DM and sepsis. T2DM contributes to chronic metabolic and immune alterations, including hyperglycaemia, low grade inflammation and comorbidity burden, which predisposes individuals to immune dysregulation and increased infection susceptibility. Upon sepsis onset, acute metabolic disturbances such as stress hyperglycaemia and glycaemic variability play a critical role in driving organ dysfunction and mortality. However, the “diabetes and obesity” paradox may further modify these relationships through hyperleptinaemia, immune priming and anti-diabetic drug effects, potentially conferring protective effects in certain populations.

The weak or negligible associations reported in some studies may have suggested that T2DM does not independently drive mortality risk in sepsis. HbA1c and admission glucose levels may not accurately reflect the real-time metabolic disturbances occurring during sepsis. Other factors (such as glycaemic control during hospitalisation, comorbidity burden, and acute disease severity) may have a greater influence on outcomes than chronic diabetic status alone. Importantly, it is not merely the presence of T2DM but the severity and chronic complications of the disease that could elevate mortality risk in sepsis patients. T2DM may only increase mortality in subpopulations with advanced disease or significant comorbidities. The “diabetes and obesity paradox”, in which patients with T2DM or obesity demonstrated lower mortality or better survival than their non-diabetic and normal-weight counterparts, may be explained by several mechanisms: (a) obesity-associated hyperleptinaemia may dampen the inflammatory response to endotoxins, (b) chronic low-grade inflammation in diabetes may prime the immune system for a more adaptive response to sepsis, and (c) some antidiabetic medications may exert anti-inflammatory or organ-protective effects. In-hospital glucose levels (rather than pre-existing diabetes or admission glucose) have been more consistently linked to mortality, highlighting the potential harm of glycaemic variability and stress hyperglycaemia. These observations could also again suggest that real-time metabolic disturbances during hospitalisation could be more critical to patient outcomes than chronic glycaemic history. Finally, various confounding factors may obscure or modify the true impact of T2DM on sepsis-related mortality. Differences in sepsis definitions across studies may contribute to these inconsistent observations. Earlier studies employing Sepsis-2 criteria, which rely on the systemic inflammatory response syndrome (SIRS), may capture a broader and more heterogenous patient population. In contrast, studies using Sepsis-3 criteria focus on organ dysfunction and tend to identify a more severely ill cohort. In fact, a substantial portion of retrospective studies in this review defined sepsis using ICD-based coding or non-standard clinical criteria, introducing further heterogeneity in case identification. Notably, the apparent “diabetes and obesity paradox” were more commonly observed in studies using administrative ICD-based definitions of sepsis, whereas studies applying Sepsis-2 or Sepsis-3 criteria generally reported neutral or inconsistent associations. This could suggest that the observed variations reported may be attributed to differences in case definitions. Therefore, future studies with careful adjustment for these variables are essential to fully elucidate the relationship between T2DM and mortality. The complex interplay between chronic metabolic alterations, immune dysfunction, acute glycaemic disturbances, and clinical outcomes in T2DM-associated sepsis is summarised in **Figure 2**.

### 3.8 Therapeutic interventions and sepsis progression in T2DM patients

Pharmacological agents targeting various immune and metabolic pathways have been investigated for their potential association with sepsis risk and outcome in patients with T2DM.

Insulin therapy is essential for managing type 1 diabetes and advanced type 2 diabetes when oral medications are insufficient 72. It helps regulate blood glucose levels and prevent complications 73. Although effective, insulin therapy requires careful monitoring to avoid hypoglycaemia 72. Exogenous insulin administration in sepsis patients resulted in elevated blood glucose and insulin levels, while concurrently reducing C-peptide concentrations and the C-peptide-to-insulin ratio compared to insulin-free sepsis patients. Notably, C-peptide level showed a negative correlation with insulin dose. The C-peptide-to-insulin ratio was inversely correlated with glycaemia and insulin dosage but positively associated with sepsis diagnosis 20.

Metformin is widely recommended as a first-line therapy for the management of T2DM, particularly in individuals who are overweight or obese 74. Beyond its glucose-lowering effects, metformin possesses anti-inflammatory and immunomodulatory properties, which may have relevance in septic conditions 46, 75. Observational studies have suggested that metformin use was associated with reduced 90-day mortality, a lower incidence of AKI, fewer major adverse kidney events at one year, and improved renal recovery in diabetic patients 32. However, these findings should be interpreted with caution as the exclusion of patients with advanced renal impairment from metformin therapy may introduce confounding by baseline health status, potentially leading to overestimation of its apparent benefits. Additionally, recent evidence indicated that early administration of metformin (500-1000 mg/day within 48-72 hours of septic shock diagnosis) was associated with reduced 90- and 365-day mortality, suggesting a possible role for early intervention 46. Notably, Shih et al. demonstrated that metformin use [both as monotherapy and in combination with other antidiabetic agents such as sulfonylureas, meglitinides, thiazolidinediones, and dipeptidyl peptidase-4 (DPP-4) inhibitors] was associated with a lower risk of hospitalisation for sepsis. Other classes of antidiabetic agents, such as sulfonylureas and meglitinides, were associated with a slightly increased risk of hospitalisation for sepsis when used as monotherapy. In contrast, thiazolidinediones showed no significant association with the risk of sepsis-related hospitalisation 40. Moreover, metformin use prior to admission has been reported to be associated with reduced 30-day sepsis-related mortality 43. Despite these results, Oh & Song did not support a significant association between metformin use and sepsis risk and 30-day mortality. This discrepancy may be partly explained by methodological differences, including the use of a lag-time approach to minimise protopathic bias by excluding patients with less than 90 days of metformin exposure. While this strategy ensured adequate exposure duration for metformin's effects to manifest, it may inadvertently exclude patients who could benefit from short-term therapy 37. Overall, the current evidence is largely observational and subject to residual confounding and bias; therefore, these findings should be interpreted as associative rather than causal, and further prospective and interventional studies are required to establish clinical efficacy and safety in septic populations.

It is important to note that although metformin is not primarily indicated for sepsis prevention, its glucose-lowering action indirectly mitigates inflammation and endothelial activation. These effects are mediated through the activation of adenosine monophosphate-activated protein kinase (AMPK) and the inhibition of NF-κB signalling pathways, ultimately downregulating the expression of pro-inflammatory cytokines 76. Furthermore, metformin-induced AMPK activation has been shown to enhance autophagy 77, which supports bacterial clearance and neutrophil function 78. The protective role of AMPK also involves the promotion of mitochondrial beta-oxidation and the induction of G1 cell cycle arrest via p53 phosphorylation 79. Given its central role in both metabolic and immune regulation, AMPK may serve as a potential therapeutic target for managing immune dysfunction in diabetic patients with sepsis.

Sodium-glucose co-transporter-2 (SGLT2) inhibitors are a class of oral antidiabetic agents that reduce glucose reabsorption in the renal proximal tubules, thereby reducing glucose toxicity and attenuating inflammation in renal tubular cells 80. A study by Hu & Lin demonstrated that SGLT2 inhibitors was associated with a reduced risk of sepsis and septic shock in T2DM patients 34. These associations have been hypothesised to relate to improvements in insulin sensitivity and reductions in inflammatory markers, such as CRP and myeloperoxidase activity (an enzyme responsible for generating ROS) 81-83. Despite the potential anti-inflammatory and metabolic benefits of SGLT2 inhibitors, their use in critically ill patients remains an ongoing concern. In particular, the risk of euglycaemic diabetic ketoacidosis (EDKA, a condition characterised by metabolic acidosis with relatively normal blood glucose levels) represents a significant safety issue in the intensive care setting 84. Sepsis may induce a catabolic metabolic state characterised by increased counter-regulatory hormone activity, including glucagon, catecholamines, and cortisol. When combined with the ketogenesis-promoting effects of SGLT2 inhibitors, this may increase the risk of EDKA despite near-normal blood glucose levels. This atypical presentation can complicate diagnosis and delay timely management in the ICU, where hyperglycaemia is often expected as a key indicator. Therefore, while SGLT2 inhibitors may be associated with potential benefits, their use in critically ill patients should be approached with caution. Further prospective and interventional studies are needed to better define their safety and clinical utility in this population.

Statins are lipid-lowering agents widely prescribed to reduce the risk of cardiovascular disease 85. They act by inhibiting 3-hydroxy-3-methylglutaryl coenzyme A (HMG-CoA) reductase, the rate-limiting enzyme in cholesterol biosynthesis thereby lowering overall cholesterol levels 86. Observational studies have reported a lower prevalence of sepsis observed among T2DM patients taking pitavastatin, pravastatin, rosuvastatin, atorvastatin, simvastatin, fluvastatin, or lovastatin. Besides, a higher cumulative exposure to statins was correlated with a lower prevalence of sepsis 41. Beyond their lipid-lowering effects, statins have been reported to exert pleiotropic actions in preclinical and experimental studies, including anti-inflammatory, immunomodulatory, and endothelial-protective effects. These include modulation of pro-inflammatory cytokine production, downregulation of Toll-like receptor signalling, and reduced leukocyte-endothelial interactions, which may be relevant in the pathophysiology of sepsis 87. Contrary to these findings, meta-analyses have reported no significant evidence supporting the protective effects of statins against T2DM or improvement in sepsis-related mortality 88, 89. While statins may be associated with a reduced risk of sepsis in patients with T2DM, current evidence remains largely associative and insufficient to support their use specifically for the prevention or treatment of sepsis. Further well-designed prospective and interventional studies are required to clarify their role in this context.

DPP-4 inhibitors are a class of oral antidiabetic drugs used to manage T2DM. By inhibiting the DPP-4 enzyme, they prolong the action of endogenous incretin hormones to enhance insulin secretion and suppress glucagon release in response to glucose 90. These medications are generally well tolerated and pose a low risk of hypoglycaemia, making them suitable for diverse patient populations 91. Linagliptin, a DPP-4 inhibitor, has been shown to attenuate systolic cardiac dysfunction, renal impairment and liver injury induced by CLP in diabetic mice. These protective effects may be attributed to the suppression of NF-κB pathway and reduced expression of inducible nitric oxide synthase (iNOS), hence dampening the inflammatory response. This proposed mechanism was further supported using IKK-16, a specific IκB kinase inhibitor, which also exhibited anti-inflammatory effects through inhibition of NF-κB activation 15. However, in clinical settings, DPP-4 inhibitors have not been significantly associated with a reduced risk of sepsis-related hospitalisation 40. The distinct outcomes between experimental and clinical studies might be attributed to several factors. Firstly, the CLP model provides a controlled and simplified representation of sepsis in animals, which may not accurately reflect the complex and heterogeneous nature of human sepsis. Secondly, differences in timing and duration of intervention might contribute. In a preclinical study, the DPP-4 inhibitor was administered one hour after sepsis induction, allowing it to exert maximal anti-inflammatory effects. In contrast, the human study included patients with varied exposure windows (including past, recent and current), thereby potentially diluting the observed treatment effects.

Emerging therapeutic strategies targeting inflammation and immune modulation have been explored in preclinical studies. A preclinical study by Frydrych et al. showed that granulocyte-macrophage colony-stimulating factor (GM-CSF) improved survival and enhanced innate immunity response in obese diabetic mice with sepsis. Although GM-CSF administration did not lead to bacterial clearance and changes in neutrophil and monocyte counts, it significantly enhanced the functional capacity of these immune cells. Specifically, GM-CSF increased the phagocytic ability of monocytes and promoted ROS generation in both neutrophils and monocytes. These findings suggested that GM-CSF showed potential as an emerging immunomodulatory agent for improving immune cell function in the management of sepsis 16.

Ulinastatin, a glycoprotein hydrolase inhibitor, has shown therapeutic potential in sepsis management by reducing mortality and improving prognosis through the inhibition of pro-inflammatory cytokines 92. A meta-analysis incorporating data from thirteen randomised controlled trials and two prospective studies further supported its efficacy in lowering all-cause mortality and improving clinical outcomes in patients with sepsis or septic shock 93. In the context of the coexistence of T2DM and sepsis, an animal study indicated that intravenous injection of 100 kU/kg of ulinastatin attenuated lung tissue damage, inflammation, and oxidative stress, therefore alleviating acute lung injury in diabetic sepsis rats 18. Despite these encouraging findings, the therapeutic benefits of ulinastatin observed in preclinical models warrant validation in well-designed, large-scale clinical trials.

## 4. Strength and limitations

This review adopts the methodology of scoping reviews to provide a comprehensive overview of the topic. By incorporating a broad range of evidence, it is well-suited for mapping the existing literature on this topic. Additionally, the inclusion of both preclinical and clinical studies offers a more holistic understanding of the current evidence. Nevertheless, this scoping review has several limitations. First, only studies published in English were included. As a result, relevant studies in other languages were excluded from our analysis and interpretation, which may have led to an incomplete representation of existing evidence. Second, two studies included in this review did not specify the type of diabetes in their study populations 38, 44. Therefore, it is unclear whether their observations were applicable to type 1 or type 2 diabetes, thus limiting the generalisability of their findings. Third, although the inclusion of a significant number of observational studies enabled broad mapping of existing literature and identification of key takeaways, these study designs are inherently prone to bias and unable to establish causality due to the presence of confounding variables. Furthermore, substantial heterogeneity was observed across the included studies, encompassing variations in sepsis and T2DM definitions and diagnostic criteria, differences in sepsis severity assessment tools (such as SOFA, LODS, APACHE II and SAPSII scoring systems), use of differing measures of glycaemic control (including HbA1c, fasting blood glucose, oral glucose tolerance test), as well as differences in reported immune and metabolic responses. This methodological and clinical heterogeneity limits direct comparability across studies and may have contributed to variability in the reported outcomes. Finally, the inclusion of grey literature may provide complementary insights. Therefore, selection biases could not be excluded for this review.

## 5. Conclusion

This scoping review highlights the complex interplay between T2DM and sepsis, where immune dysregulation, chronic inflammation and metabolic disturbances may contribute to increased susceptibility to infection and greater disease severity. While mortality outcomes remain inconsistent, emerging evidence suggested that anti-diabetic therapies such as metformin, SGLT2 inhibitors, DPP-4 inhibitors and statins have been associated with improved sepsis outcomes. However, these findings are subject to potential bias and confounding as well as should be interpreted as hypothesis-generating rather than evidence of causal benefit. Robust interventional human studies are required to determine their efficacy and safety in the context of sepsis.

An important area for future investigation is the determination of optimal glycaemic targets in septic patients with pre-existing diabetes. Chronic hyperglycaemia may induce adaptive metabolic changes at the cellular level, potentially shifting the thresholds at which hypoglycaemia-induced cellular starvation or hyperglycaemia-related toxicity occur. Consequently, glycaemic targets appropriate for non-diabetic individuals may not be directly applicable to patients with diabetes. Further studies are needed to define personalised glycaemic targets that account for baseline metabolic status and disease severity in this population. In addition, emerging immunomodulatory therapies including NF-κB inhibitors, GM-CSF and ulinastatin, have demonstrated promise in preclinical models. Nevertheless, their relevance to human disease remains uncertain. Further translational and clinical research is required before any conclusions regarding their therapeutic role can be drawn.

## Supplementary Material

Supplementary tables.

## Figures and Tables

**Table 1:** Characteristics and major findings of included experimental *in vivo* animal model studies.

Authors (Year)	Subjects	Outcomes
Frydrych et al. (2019) 16	Male C57BL/6J mice fed with high-fat diet and subjected to CLP	Metabolic dysregulation: Hyperglycaemia, hyperinsulinaemia, insulin resistance, and glucose intolerance Infection susceptibility: ↑ bacterial loads Inflammation: ↓ MIP-1α, TNF-α and IL-10 ↑ IL-6 and IFN-γ Immune dysregulation: ↓ neutrophils and monocytes ↓ phagocytic ability and ROS generation ↓ functional ability of neutrophils (↓ *Axl* and *Mertk* genes) Mortality: ↑ mortality rate due to higher weight loss Treatment effect of GM-CSF (10 ng): ↑ survivability no change in bacterial counts no change in neutrophil and monocyte numbers in bone marrow and spleen ↑ phagocytic ability in monocyte ↑ ROS generation in neutrophils and monocytes
Al Zoubi et al. (2018) 15	Male C57BL/6 mice fed with high-fat diet and subjected to CLP	Metabolic dysregulation: Impaired glucose tolerance and insulin resistance ↑ fasting blood glucose and plasma insulin Inflammation: ↑ TNF-α, IL-6, keratinocyte-derived chemokine, IL-10, and MPO ↑ NAG ↓ Akt phosphorylation (Ser473) ↑ phosphorylation of IKKα/β, IκBα, p65 translocation and iNOS expression Organ function: Renal dysfunction (↑ creatinine, urea, ALT, and urine albumin-to-creatinine ratio) Cardiac dysfunction (↓ ejection fraction, fractional shortening, and functional area change) Therapeutic effects of linagliptin (10 mg/kg, i.v.) and IKK-16 (1 mg/kg, i.v.): ↑ ejection fraction, fractional shortening, and functional area change ↓ ALT, creatinine, and urea ↓ IL-6, keratinocyte-derived chemokine, IL-10, MPO, and NAG ↑ Akt phosphorylation (Ser473) ↓ phosphorylation of IKKα/β, IκBα, p65 translocation and iNOS expression
Jacob et al. (2008) 17	Male Goto-Kakizaki rats subjected to CLP	Metabolic dysregulation: ↑ glucose Inflammation: ↑ IL-6 and IL-10
Jin et al. (2022) 18	Male Sprague-Dawley rats fed with a high-fat diet, injected with STZ and LPS	Metabolic dysregulation: ↑ glucose Inflammation: ↑ IL-1β, IL-18, TNF-α, HIF-1α, and TLR4 Organ function: ↑ microvascular permeability Severe lung tissue deformation, collapsed alveolar walls, widened lung interstitium, atelectasis, inflammatory cell infiltration, red blood cells present in lung interstitium and alveoli, exfoliated epithelial cells in bronchi, telangiectasia, loss of lung bubble cavities Oxidative stress: ↑ MDA, ↓ SOD Therapeutic effects of ulinastatin (100 kU/kg, i.v.): ↓ IL-1β, IL-18, TNF-α, HIF-1α, and TLR4 ↓ MDA, ↑ SOD ↓ microvascular permeability ↓ lung tissue damage
Weng et al. 2024 47	Male C57BL/6 mice fed with high-fat diet, injected with STZ and CLP Bioinformatics analysis: Samples from Gene Expression Omnibus database (T2DM and sepsis patients)	Inflammation: ↑ MMP-8 and CD177 in liver and kidneys ↑ MMP-8 in cardiac tissue ↑ MMP-8, CD177, and S100A12 expressions Organ function: ↑ LDH, AST, ALT, creatinine, and BUN and ↓ albumin, suggesting severe organ damage Lipid deposition, cell swelling, necrosis, disrupted tissue architecture and inflammation in liver and kidneys Bioinformatics analysis from T2DM and sepsis patients: ↑ MMP-8, CD177 and S100A12 ↑ neutrophils, plasma cells, monocytes, M2 macrophages, mast cells and eosinophils ↓ CD8 T cell, memory B cells, dendritic cells and resting natural killer cells

**Abbreviations:** Akt: protein kinase B; ALT: alanine aminotransferase; AST: aspartate aminotransferase; *Axl*: Anexelekto; BUN: blood urea nitrogen; CD177: cluster of differentiation-177; CD8: cluster of differentiation 8; CLP: caecal ligation puncture; GM-CSF: granulocyte-macrophage colony-stimulating factor; HIF-1α: hypoxia-inducible factor-1 alpha; IFN-γ: interferon-gamma; IKK-16: selective IκB inhibitor; IKKα/β: Inhibitor of nuclear factor-kappa B kinase subunit alpha and beta; IκBα: inhibitor of nuclear factor-kappa B alpha; IL-1β: interleukin-1 beta; IL-6: interleukin-6; IL-10: interleukin-10; IL-18: interleukin-18; iNOS: inducible nitric oxide synthase; i.v.: intravenous; LDH: lactate dehydrogenase; MDA: malondialdehyde; *Mertk*: MER tyrosine kinase; MIP-1α: macrophage inflammatory protein-1 alpha; MMP-8: matrix metalloproteinase-8; MPO: myeloperoxidase; NAG: N-acetyl-β-D-glucosaminidase; p65: nuclear factor-kappa B p65 subunit; ROS: reactive oxygen species; S100A12: S100 calcium-binding protein A-12; SOD: superoxide dismutase; TLR4: toll-like receptor 4; TNF-α: tumour necrosis factor-alpha.

**Table 2:** Characteristics of included prospective and retrospective observational studies.

Author (Year)	Study years	Country	Study design	Population & sample size	Sepsis definition	T2DM definition
Balintescu et al. (2022) 19	2005-2015	Sweden	Prospective observational	T2DM patients aged ≥18 years (n=502,871)	ICD-10-CM codes	Treatment with diet with or without additional treatment with noninsulin glucose-lowering drugs or treatment with insulin
Bitker et al. (2019) 20	2016-2018	Australia	Prospective observational	T2DM patients aged ≥ 18 years (n = 31)	Sepsis-3 criteria	Pre-existing clinician-diagnosed T2DM; diagnostic criteria not specified
Bloch et al. (2021) 21	2015-2016	Israel	Prospective observational	Sepsis patients with and without T2DM aged ≥ 18 years (n = 37)	Sepsis-2 criteria	Pre-existing clinician-diagnosed T2DM; diagnostic criteria not specified
Gornik et al. (2007) 22	2003-2005	Croatia	Prospective observational	Sepsis patients with T2DM aged ≥ 18 years (n = 286)	Sepsis-2 criteria	2003 American Diabetes Association criteria
Gornik et al. (2010) 23	1998-2003	Croatia	Prospective observational	Sepsis patients aged ≥ 18 years (n = 1,843)	Sepsis-2 criteria	2008 American Diabetes Association criteria
Jia et al. (2016) 24	2014-2015	China	Prospective observational	Sepsis patients with and without T2DM aged ≥ 18 years (n = 165)	Sepsis-2 criteria	2013 American Diabetes Association criteria
Perl et al. (2018) 25	2015-2016	Israel	Prospective observational	Sepsis patients with and without T2DM aged ≥ 18 years (n = 37)	Sepsis-2 criteria	Pre-existing clinician-diagnosed T2DM; diagnostic criteria not specified
Thockchom et al. (2023) 26	2022	India	Prospective observational	Sepsis and septic shock patients with T2DM aged ≥ 18 years (n = 32)	Sepsis-3 criteria	2016 American Diabetes Association criteria
Yagmur et al. (2019) 27	2006-2011	Germany	Prospective observational	Sepsis patients with T2DM aged ≥ 18 years (n = 218)	Sepsis-3 criteria	Pre-existing clinician-diagnosed T2DM; diagnostic criteria not specified
Chang et al. (2012) 28	1998-2008	Taiwan	Retrospective observational	Sepsis patients with and without T2DM aged ≥ 18 years (n = 16,497)	Documented infection, either bacterial or fungal, plus at least one acute organ dysfunction based on ICD-9-CM codes	ICD-9-CM code 250.X present in the outpatient file ≥ 3 times in the year before severe sepsis was diagnosed; T2DM criteria not specified
D'Almeida et al. (2021) 29	2020	Samoa	Retrospective observational	T2DM patients aged ≥ 18 years (n = 100)	Sepsis-3 criteria	Recorded diagnosis of T2DM (duration ≥ 5 years and on more than one diabetes medication) in hospital records
de Miguel-Yanes et al. (2015) 30	2008-2012	Spain	Retrospective observational	Sepsis patients with and without T2DM aged ≥ 18 years (n = 217,280)	ICD-9-CM codes	ICD-9-CM codes 250.x0 and 250.x2
Feng & Zhang (2023) 31	2019-2022	China	Retrospective observational	Sepsis patients with T2DM aged ≥ 18 years (n = 36)	Not reported	Not reported
Gómez et al. (2022) 32	2008-2014	United States of America	Retrospective observational	Sepsis patients with T2DM aged ≥ 18 years (n = 14,847)	Sepsis-3 criteria	ICD-9-CM codes 250.x0 and 250.x2
Hsieh et al. (2019) 33	1999-2012	Taiwan	Retrospective observational	Sepsis patients with and without T2DM (n = 19,719)	ICD-9-CM code 038 plus infection diagnosis and antibiotic use	ICD-9-CM codes in the NHIRD LHDB, with diagnosis confirmed ≥ 1 year before the patient's first sepsis hospitalisation
Hu & Lin (2024) 34	2016-2019	Taiwan	Retrospective observational	T2DM patients aged ≥ 20 years (n = 61,496)	ICD-9-CM and ICD-10-CM codes in the NHIRD, with ≥2 outpatient claims or ≥1 inpatient claim for sepsis/septic shock	ICD-9-CM and ICD-10-CM codes in the NHIRD
Li et al. (2024) 35	2024	United States of America	Retrospective observational	Sepsis patients with T2DM aged > 65 years (n = 1,489)	Sepsis-3 criteria	ICD-9-CM codes from the MIMIC-IV database
Li et al. (2022) 36	2017-2019	China	Retrospective observational	Sepsis patients with T2DM aged ≥ 18 years (n = 250)	Sepsis-3 criteria	2016 American Diabetes Association criteria
Oh & Song (2020) 37	2010-2015	South Korea	Retrospective observational	T2DM patients aged ≥ 18 years (n = 77,337)	ICD-10-CM codes	ICD-10-CM codes
Petrovici et al. (2013)* 38	2008-2010	Romania	Retrospective observational	Sepsis patients with DM aged ≥ 18 years (n = 445)	Clinician-diagnosed sepsis with microbiologically confirmed or suspected infection; no formal consensus criteria specified.	American Diabetes Associations criteria (guideline year not specified)
Sathananthan et al. (2020) 39	2001-2012	United States of America	Retrospective observational	Sepsis patients with and without T2DM aged ≥ 16 years (n = 445)	ICD-9-CM codes from the MIMIC-IV database	ICD-9-CM codes 250.x0 and 250.x2 from the MIMIC-IV database
Shih et al. (2015) 40	2010-2012	Taiwan	Retrospective observational	T2DM patients aged ≥ 18 years (n = 86,030)	ICD-9-CM codes plus prescription of antibiotics	ICD-9-CM code in the NHIRD, two ambulatory visits with a diagnosis of diabetes or use of any antidiabetic drug
Sun et al. (2023) 41	2008-2021	Taiwan	Retrospective observational	T2DM patients aged ≥40 years (n = 812,420)	ICD-9-CM and ICD-10-CM codes in the NHIRD	ICD-9-CM and ICD-10-CM codes in the NHIRD
Xin et al. (2022) 42	2015-2021	China	Retrospective observational	Sepsis patients with T2DM aged ≥ 18 years (n = 406)	Sepsis-3 criteria	2022 American Diabetes Association
Yang et al. (2021) 43	2001-2012	United States of America	Retrospective observational	Sepsis patients with T2DM aged ≥ 18 years (n = 2,883)	Sepsis-3 criteria	ICD-9-CM codes from the MIMIC-III database
Yang et al. (2011)* 44	2004-2008	Singapore	Retrospective observational	Sepsis patients with and without DM aged ≥ 18 years (n = 9,221)	ICD-9-AM codes	ICD-9-AM code 250
Lee et al. (2025) 45	2009-2020	South Korea	Retrospective observational	T2DM patients aged ≥ 20 years (n = 3,863,323)	ICD-10-CM codes	ICD-10 code and/or prescription records for oral antidiabetic drugs and short- or long-acting insulin within 3 months after screening
Jin et al. (2025) 46	2016-2022	South Korea	Retrospective observational	Sepsis patients with T2DM aged ≥ 18 years (n = 320)	Sepsis-3 criteria	Pre-existing clinician-diagnosed T2DM; diagnostic criteria not specified

**Notes:** * indicates studies that did not specify the type of diabetes. **Abbreviations:** ADA, American Diabetes Association; DM, diabetes mellitus; ICD-9-AM, International Classification of Diseases, Ninth Revision, Australian Modification; ICD-9-CM, International Classification of Diseases, Ninth Revision, Clinical Modification; ICD-10-CM, International Classification of Diseases, Tenth Revision, Clinical Modification; LHDB, Longitudinal Health Database; MIMIC-III, Medical Information Mart for Intensive Care III; MIMIC-IV, Medical Information Mart for Intensive Care IV; NHIRD, National Health Insurance Research Database; T2DM, type 2 diabetes mellitus.

**Table 3:** Outcomes of included prospective observational studies.

Author (Year)	Outcome measure definitions	Glycaemic parameter classification	Outcomes	Reported effect sizes
Balintescu et al. (2022) 19	Sepsis risk: time from T2DM diagnosis to first recorded sepsis All-cause mortality: death from any cause throughout the index hospital admission	Pre-sepsis glycaemic control (HbA1c)	U-shaped association between HbA1c and sepsis risk (↓ sepsis risk at HbA1c ~53 mmol/mol; ↑ sepsis risk at HbA1c <43 mmol/mol and >82 mmol/mol). No significant association between HbA1c and mortality. ↑ comorbidities including stroke, heart failure, cancer, renal dialysis or transplantation, lung disease, liver disease, haematological disease and immunological deficiency in sepsis patients.	Sepsis risk according to HbA1c level: <43 mmol/mol: HR=1.15 (95% CI 1.07-1.24) 53-62 mmol/mol: HR=0.93 (95% CI 0.87-0.99) >82 mmol/mol: HR=1.52 (95% CI 1.37-1.68) Mortality depending on HbA1c level: <43 mmol/mol: HR=1.03 (95% CI 0.92-1.15) 48-52 mmol/mol: HR=1.00 >82 mmol/mol: HR=1.06 (95% CI 0.90-1.24)
Bitker et al. (2019) 20	C-peptide to insulin ratio: ratio of serum C-peptide to serum insulin levels Glucose measurement: arterial blood glucose	Acute hyperglycaemia	Insulin exposure led to ↑ glucose and insulin levels and ↓ C-peptide and C-peptide to insulin ratio. C-peptide to insulin ratios was positively correlated with sepsis diagnosis.	Not available
Bloch et al. (2021) 21	GLP-1 measurement: plasma GLP-1 concentrations (total GLP: isoforms 7-36 and 9-36; active GLP: isoforms 7-36 and 7-37) quantified via ELISA PCT measurement: plasma PCT levels quantified via ELISA	Not assessed	↑ total and active GLP-1 in diabetic as compared to non-diabetic patients. ↓ PCT in diabetic as compared to non-diabetic patients.	Not available
Gornik et al. (2007) 22	Hospital mortality: death throughout the index hospital admission	Pre-sepsis glycaemic control (HbA1c) and acute hyperglycaemia	↑ urinary tract, respiratory system, biliary tract or skin and soft tissue infections among sepsis patients with T2DM. HbA1c levels were positively correlated with ↑ hospital mortality and hospitalisation. No significant association between blood glucose and hospital mortality or hospitalisation.	Hospital mortality among sepsis patients with T2DM: OR=1.358; 95% CI 1.171-1.574)
Gornik et al. (2010) 23	Fasting hyperglycaemia measurement: having fasting glucose levels of 100-125 mg/dl (5.6-6.9 mmol/l) Impaired glucose tolerance measurement: having glucose levels of 140-199 mg/dl (7.8-11.1 mmol/l) 2 hours post glucose load T2DM development: new onset of T2DM during ≥5-year follow-up	Pre-sepsis glycaemic control (HbA1c)	↑ severe sepsis and septic shock in patients with hyperglycaemia. Hyperglycaemia was associated with ↑ APACHE II score, SOFA score, and hospital stay.	Incidence of fasting hyperglycaemia/impaired glucose tolerance: RR=1.84 (95% CI 1.18-2.89) T2DM development: RR=4.29 (95% CI 1.35-13.64)
Jia et al. (2016) 24	PD-1^+^ T cell expression: Percentage of PD-1⁺CD4⁺ and PD-1⁺CD8⁺ T cells from peripheral venous blood collected on admission using flow cytometry. Disease severities: APACHE II and SOFA scores within 24 hours of admission 28-day mortality: death from any cause occurring within 28 days after admission for severe sepsis	Pre-sepsis glycaemic control (HbA1c) and acute hyperglycaemia	↑ HbA1c and blood glucose levels in diabetic sepsis patients. No difference in PD-1^+^ T cell expression between diabetic and non-diabetic sepsis patients. Percentage of PD-1^+^ CD4^+^ T cells and PD-1^+^ CD8^+^ T cells were positively correlated with APACHE II and SOFA scores in patients with severe sepsis (with and without T2DM). No difference in white blood cell counts between diabetic and non-diabetic sepsis patients. No difference in SOFA and APACHE II scores between diabetic and non-diabetic sepsis patients. No difference in 28-day mortality between diabetic and non-diabetic sepsis patients.	Correlation between PD-1^+^ CD4^+^ T cells and APACHE II scores: r=0.627 Correlation between PD-1^+^ CD8^+^ T cells and APACHE II scores: r=0.649 Correlation between PD-1^+^ CD4^+^ T cells and SOFA scores: r=0.566 Correlation between PD-1^+^ CD8^+^ T cells and SOFA scores: r=0.556
Perl et al. (2018) 25	Endogenous GLP-1 parameters: plasma GLP-1 concentrations (total GLP: isoforms 7-36 and 9-36; active GLP: isoforms 7-36 and 7-37) quantified via ELISA Inflammatory marker measurement: serum CRP concentrations	Not assessed	Surviving patients had ↑ active GLP-1. ↑ CRP in sepsis patients with T2DM than without T2DM. ↑ CRP in non-surviving than surviving sepsis patients with T2DM.	Not available
Thockchom et al. (2023) 26	Glycaemic measures: fasting blood glucose, postprandial blood sugar, and HbA1c Cardiac markers: NT-proBNP Diastolic dysfunction: heart's ability to relax, assessed via echocardiography, graded according to American Society of Echocardiography 2009 guidelines 28-day mortality: death from any cause occurring within 28 days after admission for sepsis	Pre-sepsis glycaemic control (HbA1c) and acute hyperglycaemia	↑ fasting blood glucose, postprandial blood sugar, and HbA1c in diabetic sepsis, but no significant association with mortality. ↑ NT-proBNP in T2DM patients with sepsis and septic shock. ↑ left ventricular diastolic dysfunction was associated with early mortality. ↑ community- and hospital-acquired pneumonia, urinary tract infections, gastrointestinal infections, skin and soft tissue infections among sepsis patients with T2DM. Dominating pathogens include *Acinetobacter baumannii*, *Escherichia coli*, *Klebsiella spp.*, *Pseudomonas spp.*, and *Streptococcus pneumoniae.*	Not available
Yagmur et al. (2019) 27	CTRP1 measurements: plasma CTRP1 quantified via ELISA	Pre-sepsis glycaemic control (HbA1c)	↑ levels of CTRP1 in diabetic sepsis patients. CTRP1 was positively correlated with HbA1c. CTRP1 was correlated with IL-6, PCT, CRPand suPAR levels. CTRP1 was correlated with creatinine, GFR-cystatin C, urea and cystatin C. CTRP1 was correlated with bilirubin, γ-glutamyltransferase and and alkaline phosphatase.	Correlation of CTRP1 and HbA1c: r=0.301 Correlation of CTRP1 and IL-6: r=0.317 Correlation of CTRP1 and PCT: r=0.414 Correlation of CTRP1 and CRP: r=0.238 Correlation of CTRP1 and suPAR: r=0.279 Correlation of CTRP1 and creatinine: r=0.283 Correlation of CTRP1 and GFR-cystatin C: r=-0.291 Correlation of CTRP1 and urea: r=0.324 Correlation of CTRP1 and cystatin C: r=0.287 Correlation of CTRP1 and bilirubin: r=0.422 Correlation of CTRP1 and γ-glutamyltransferase: r=0.243 Correlation of CTRP1 and alkaline phosphatase: r=0.211

**Abbreviations:** APACHE II, Acute Physiology and Chronic Health Evaluation II; CI, confidence interval; CRP, C-reactive protein; CTRP1, C1q/TNF-related protein-1; ELISA, enzyme-linked immunosorbent assay; GFR, glomerular filtration rate; GLP-1, glucagon-like peptide-1; HbA1c, glycated haemoglobin; HR, hazard ratio; IL-6, interleukin-6; NT-proBNP, N-terminal pro-B-type natriuretic peptide; OR, odds ratio; PCT, procalcitonin; PD-1, programmed cell death protein-1; RR, relative risk; SOFA, Sequential Organ Failure Assessment; suPAR, soluble urokinase plasminogen activator receptor; T2DM, type 2 diabetes mellitus.

**Table 4:** Outcomes of included retrospective observational studies.

Author (Year)	Outcome measure definitions	Glycaemic parameter	Outcomes	Reported effect sizes
Chang et al. (2012) 28	Organ dysfunction: relative risk of developing acute organ dysfunction (AKI, respiratory, haematological or hepatic) based on reported ICD-9-CM codes 90-day in hospital mortality: death identified as either “death” or “discharged in terminally ill state” within 90 days after sepsis admission	Not assessed	↑ infections of the genitourinary tract, gastrointestinal, skin, soft tissue and bone among sepsis patients with T2DM. Sepsis patients with T2DM had ↑ risk of developing AKI and underwent haemodialysis. Sepsis patients with T2DM had ↓ risk of developing respiratory, haematological and hepatic dysfunction 90-day in-hospital mortality between diabetics with severe sepsis did not show significant difference compared to non-diabetics.	AKI development: RR=1.54 (95% CI 1.44-1.63) Respiratory dysfunction development: RR=0.96 (95% CI 0.94-0.97) Haematological dysfunction development: RR= 0.70 (95% CI 0.56-0.89) Hepatic dysfunction development: RR=0.77 (95% CI 0.63-0.93) 90-day hospital mortality: HR=0.979 (95% CI 0.908-1.055)
D'Almeida et al. (2021) 29	Glycaemic control measures: random blood glucose and HbA1c levels at admission Infection/inflammation marker: WBC count at admission	Pre-sepsis glycaemic control (HbA1c) and acute hyperglycaemia	↑ skin and soft tissue infections, respiratory infection and urinary tract infection among sepsis patients with T2DM. No correlation between random blood glucose and HbA1c with the stages of sepsis. ↑ WBCs in sepsis, followed by severe sepsis and septic shock.	Random blood glucose and HbA1c levels among sepsis patients with T2DM: Mean=17.60 (SD=8.559) Mean=10.72 (SD=2.605) Random blood glucose and HbA1c levels among severe sepsis patients with T2DM: Mean= 20.38 (SD=9.683) Mean=9.986 (SD=2.516) Random blood glucose and HbA1c levels among septic shock patients with T2DM: Mean=15.98 (SD=5.801) Mean=8.980 (SD=1.499) WBC count among sepsis patients with T2DM: Mean=17.54 (SD=8.083) WBC count among severe sepsis patients with T2DM: Mean= 16.67 (SD=9.350) WBC count among septic shock patients with T2DM: Mean=15.99 (SD=6.430)
de Miguel-Yanes et al. (2015) 30	In-hospital mortality: deaths during admission Length of hospital stay: duration of hospitalisation	Not assessed	↓ in-hospital mortality in sepsis patients with T2DM. ↓ length of hospital stays in sepsis patients with T2DM.	In-hospital mortality among sepsis patients with T2DM: OR=0.88 (95% CI 0.86-0.90)
Feng & Zhang (2023) 31	Not available	Not available	↑ incidence of occult primary infection sites and severe infections among sepsis patients with T2DM. ↑ detection of antibiotic-resistant bacterial strains (ESBL-producing *Escherichia coli* and MRSA). Sepsis patients with T2DM exhibited poor blood glucose control.	Not available
Gómez et al. (2022) 32	90-day mortality: death from any cause within 90 days from sepsis diagnosis Severe AKI: development of severe AKI 24 hours before sepsis diagnosis, hospital discharge or death (whichever occurred first) AKI recovery: recovery of kidney function following AKI	Not assessed	Exposure to metformin was associated with ↓ 90-day mortality. Exposure to metformin was associated ↓ severe AKI and ↑ renal recovery.	90-day mortality among patients with T2DM taking metformin: OR = 0.46 (95% CI 0.35-0.60) Severe AKI among patients with T2DM taking metformin: OR = 0.75 (95% CI 0.62-0.90) Renal recovery among patients with T2DM taking metformin: OR = 6.43 (95% CI 3.42-12.1)
Hsieh et al. (2019) 33	Hospital mortality: death during the index hospitalisation	Pre-sepsis glycaemic control (HbA1c) and acute hyperglycaemia	↑ mortality among sepsis patients with T2DM. ↑ hospital mortality of sepsis as DCSI scores increased in sepsis patients with T2DM. ↑ initial creatinine levels, prevalence of haemodialysis and respiratory support in sepsis patients with T2DM.	Hospital mortality among sepsis patients with T2DM: OR = 1.14 (95% CI 1.10-1.19) Mortality among sepsis patients with T2DM with a DCSI score of ≥5: OR = 1.77 (95% CI 1.61-1.96)
Hu & Lin (2024) 34	Sepsis incidence: new diagnosis of sepsis after the index date	Not assessed	↑ DCSI was associated with ↑ risk of sepsis/septic shock. Taking SGLT2 inhibitor for ≥90 days was associated with ↓ sepsis/septic shock in T2DM patients.	Sepsis risk among T2DM patients with increased DCSI: HR = 1.52 (95% CI 1.37-1.68) Sepsis risk among T2DM patients taking SGLT2 inhibitor for ≥90 days: HR=0.36 (95% CI 0.34-0.39)
Li et al. (2024) 35	28-day mortality: death occurring within 28 days of ICU admission	Not assessed	Age, length of ICU stays, metastatic solid tumour, SOFA score, vasopressin use, LODS, mean blood pressure and BUN were identified as significant 28-day mortality predictors among sepsis patients with T2DM. ↑ WBCs and prevalence of AKI, MI, CHF, severe liver disease and metastatic solid tumours among deceased sepsis patients with T2DM. ↑ dialysis, ventilation, vasopressin, adrenaline, noradrenaline and neuroblock use among deceased sepsis patients with T2DM. ↑ LODS, SOFA and SAPSII scores, BUN, creatinine, potassium, anion gap and heart rate among deceased sepsis patients with T2DM.	Age and mortality: OR=1.055 (95% CI 1.035-1.076) Length of ICU stay and mortality: OR = 0.962 (95% CI 0.940-0.984) Metastatic solid tumour and mortality: OR = 2.902 (95% CI 1.594-5.283) SOFA score and mortality: OR = 1.185 (95% CI 1.121-1.253) Vasopressin use and mortality: OR = 2.974 (95% CI 1.998-4.427) LODS score and mortality: OR = 1.075 (95% CI 1.012-1.142) Mean blood pressure and mortality: OR = 1.013 (95% CI 1.006-1.021) BUN and mortality: OR=1.007 (95% CI 1.002-1.012)
Li et al. (2022) 36	Gene expression: relative MCP-1 mRNA expression levels in peripheral blood mononuclear cells, quantified using RT-qPCR and genotype distribution of MCP-1 rs1024611 polymorphism, determined using the SNaPshot assay Inflammatory marker measurement: plasma TNF-α, IL-6, and IL-1β levels quantified using ELISA	Not assessed	↑ frequencies of rs1024611 AG/GG genotypes and G allele in T2DM patients with sepsis. ↑ MCP-1 and TNF-α in GG genotypes of in T2DM patients with sepsis compared to AA or GA genotypes ↑ TNF-α, IL-6, and IL-1β in T2DM patients with sepsis. ↑ respiratory tract, gastrointestinal tract, and bloodstream infections among sepsis patients with T2DM, with *Acinetobacter baumannii*, *Klebsiella pneumoniae*, and *Staphylococcus aureus* as dominating pathogens.	AG genotype in T2DM patients with sepsis: OR = 2.44 (95% CI 1.31-4.53) GG genotype in T2DM patients with sepsis: OR = 2.12 (95% CI 1.05-4.28) G allele in T2DM patients with sepsis: OR = 1.43 (95% CI 1.02-2.01)
Oh & Song (2020) 37	Sepsis development: new sepsis diagnosis reported during study period 30-day mortality: death from any cause within 30 days after sepsis diagnosis	Not assessed	No association between metformin use and sepsis risk. No association between metformin use and 30-day mortality.	Metformin exposure and sepsis risk: OR = 0.92 (95% CI 0.82-1.03) Metformin exposure and mortality: OR = 0.94 (95% CI 0.75-1.17)
Petrovici et al. (2013) 38	Disease severity: Charlson and SAPSII scores	Not assessed	↑ Charlson and SAPSII scores in sepsis patients with DM. ↑ chronic liver disease and altered consciousness among sepsis patients with DM.	Not available
Sathananthan et al. (2020) 39	Hospital mortality: death occurring during hospitalisation in which patients were treated for sepsis Glucose measurements: blood glucose levels taken on admission and throughout hospital stay	Acute hyperglycaemia	↑ prevalence of congestive heart failure, hypertension, end-stage renal disease, and chronic alcoholism in sepsis patients with T2DM. ↑ AKI and required acute haemodialysis in sepsis patients with T2DM. No difference in gram-positive, gram-negative, anaerobic, atypical, or mixed infections between sepsis patients with and without T2DM. T2DM and admission glucose were not significantly associated with mortality among sepsis patients with T2DM. Mean glucose during hospital stay was positively associated with mortality among sepsis patients with T2DM. Older age, presence of cirrhosis, transplant status, and severe sepsis were associated with ↑ mortality.	T2DM and mortality among sepsis patients: OR = 0.89 (95% CI 0.70-1.13) Admission glucose and mortality among sepsis patients: OR = 0.82 (95% CI 0.62-1.07) Hospital glucose and mortality among sepsis patients: OR = 2.47 (95% CI 1.76-3.47)
Shih et al. (2015) 40	Sepsis incidence: hospitalisation for sepsis	Not assessed	Metformin was associated with ↓ sepsis risk. Sulfonylurea and meglitinide were associated with ↑ sepsis risk. No association between DPP-4 inhibitors and thiazolidinedione with sepsis risk. Combination of metformin with sulfonylurea, meglitinide, DPP-4 inhibitors or thiazolidinedione were associated with ↓ sepsis risk.	Metformin and sepsis risk: OR = 0.80 (95% CI 0.77-0.83) Sulfonylurea and sepsis risk: OR = 1.06; (95% CI 1.03-1.10) Sulfonylurea + metformin and sepsis risk: OR = 0.72 (95% CI 0.69-0.75) Meglitinide and sepsis risk: OR = 1.32 (95% CI 1.25-1.40) Meglitinide + metformin and sepsis risk: OR = 0.82 (95% CI 0.71-0.96) DPP-4 inhibitor and sepsis risk: OR = 1.01 (95% CI 0.95-1.06) DPP-4 inhibitors + metformin and sepsis risk: OR = 0.65 (95% CI 0.55-0.78) Thiazolidinedione and sepsis risk: OR = 0.95 (95% CI 0.89-1.01) Thiazolidinedione + metformin and sepsis risk: OR = 0.51 (95% CI 0.41-0.64)
Sun et al. (2023) 41	Sepsis development: first hospitalisation for sepsis during follow-up period	Not assessed	The use of statin was associated with ↓ prevalence of sepsis. The use of pitavastatin, pravastatin, rosuvastatin, atorvastatin, simvastatin, fluvastatin and lovastatin were associated with ↓ prevalence of sepsis. ↑ cumulative statin exposure correlated with ↓ prevalence of sepsis.	Statins and sepsis prevalence: HR = 0.37 (95% CI 0.35-0.38) Pitavastatin and sepsis prevalence: HR = 0.09 (95% CI 0.05-0.14) Pravastatin and sepsis prevalence: HR = 0.32 (95% CI 0.31-0.34) Rosuvastatin and sepsis prevalence: HR = 0.34 (95% CI 0.32-0.36) Atorvastatin and sepsis prevalence: HR = 0.35 (95% CI 0.32-0.37) Simvastatin and sepsis prevalence: HR = 0.37 (95% CI 0.34-0.39) Fluvastatin and sepsis prevalence: HR = 0.42 (95% CI 0.38-0.44) Lovastatin and sepsis prevalence: HR = 0.54 (95% CI 0.51-0.56) Cumulative statin exposure and sepsis prevalence: HR = 0.17 (95% CI 0.15, 0.19)
Xin et al. (2022) 42	Major adverse kidney events within 30 days: occurrence of all-cause mortality, new requirement for renal replacement therapy and/or persistent renal dysfunction within 30 days after study inclusion or at hospital discharge	Not assessed	Hypotension, thrombocytopenia, dyslipidaemia and early renal dysfunction are key determinants of major adverse kidney event within 30 days in sepsis patients with T2DM.	MAP and major adverse kidney events within 30 days: OR = 0.928 (95% CI 0.906-0.950) Platelet and major adverse kidney events within 30 days: OR = 0.995 (95% CI 0.992-0.999) HDL and major adverse kidney events within 30 days: OR = 0.009 (95% CI 0.002-0.036) ApoE and major adverse kidney events within 30 days: OR = 0.988; 95% CI 0.979-0.997) Cystatin C and major adverse kidney events within 30 days: OR = 1.960 (95% CI 0.360-2.826)
Yang et al. (2021) 43	30-day mortality: death occurring within 30 days of hospital admission	Not assessed	Preadmission metformin use was associated with ↓ 30-day mortality in sepsis patients with T2DM.	Metformin and mortality among sepsis patients with T2DM: HR = 0.61 (95% CI 0.46-0.81)
Yang et al. (2011) 44	Infection sources: anatomical site of infection Organ dysfunction: reported organ dysfunction using diagnostic codes	Not assessed	↑ frequency of renal, skin, soft tissue and bone infections in sepsis patients with DM. ↓ frequency of respiratory, gastrointestinal, cardiovascular, and neurological infections in sepsis patients with DM. ↑ renal and metabolic dysfunction among sepsis patients with DM. ↓ respiratory, hepatic, and haematological dysfunction among sepsis patients with DM.	Not available
Lee et al. (2025) 45	Sepsis incidence: new-onset sepsis occurred during the follow-up period	Acute hyperglycaemia	Impaired fasting glucose was associated with ↑ sepsis risk. Individuals having T2DM for ≥5 years had ↑ sepsis risk.	Impaired fasting glucose and sepsis risk: HR = 1.03 (05% CI 1.01-1.05) Having T2DM for ≥5 years and sepsis risk: HR = 1.82 (95% CI 1.77-1.87)
Jin et al. (2025) 46	90-day all-cause mortality: death from any cause within 90 days after hospital admission for septic shock 365-day all-cause mortality: death from any cause within 365 days after hospital admission for septic shock	Not assessed	Metformin administration within 48 and 72 hours was associated with ↓ 90- and 365-day mortality. 500-1000 mg/day metformin was associated with ↓ 90- and 365-day mortality.	Metformin within 48 hours and 90- and 365-day mortality: HR = 0.371 (95% CI 0.153-0.900) HR = 0.453 (95% CI 0.219-0.937) Metformin within 72 hours and 90- and 365-day mortality: HR = 0.433 (95% CI 0.235-0.797) HR = 0.450 (95% CI 0.264-0.767) Metformin does of 500-1000 mg/day and 90- and 365-day mortality: HR = 0.311 (95% CI 0.115-0.840) HR = 0.384 (95% CI 0.163-0.907)

**Abbreviations:** AKI, acute kidney injury; ApoE, apolipoprotein E; BUN, blood urea nitrogen; CHF, congestive heart failure; CI, confidence interval; DCSI, Diabetes Complications Severity Index; DM, diabetes mellitus; DPP-4, dipeptidyl peptidase-4; ELISA, enzyme-linked immunosorbent assay; ESBL, extended-spectrum beta-lactamase; HbA1c, glycated haemoglobin; HDL, high-density lipoprotein; HR, hazard ratio; ICU, intensive care unit; ICD-9-CM, International Classification of Diseases, Ninth Revision, Clinical Modification; IL-1β, interleukin-1 beta; IL-6, interleukin-6; LODS, Logistic Organ Dysfunction System; MAP, mean arterial pressure; MCP-1, monocyte chemoattractant protein-1; MI, myocardial infarction; MRSA, methicillin-resistant *Staphylococcus aureus*; OR, odds ratio; RR, relative risk; RT-qPCR, reverse transcription quantitative polymerase chain reaction; SAPSII, Simplified Acute Physiology Score II; SD, standard deviation; SGLT2, sodium-glucose cotransporter-2; SOFA, Sequential Organ Failure Assessment; T2DM, type 2 diabetes mellitus; TNF-α, tumour necrosis factor alpha; WBC, white blood cell.

## References

[1] Arora J, Mendelson AA, Fox-Robichaud A (2023). Sepsis: network pathophysiology and implications for early diagnosis. American journal of physiology Regulatory, integrative and comparative physiology.

[2] Rudd KE, Johnson SC, Agesa KM, Shackelford KA, Tsoi D, Kievlan DR (2020). Global, regional, and national sepsis incidence and mortality, 1990-2017: analysis for the Global Burden of Disease Study. Lancet (London, England).

[3] Mahapatra S, Heffner AC (2025). Septic Shock. Treasure Island (FL): StatPearls Publishing.

[4] Jacobi J (2022). The pathophysiology of sepsis-2021 update: Part 1, immunology and coagulopathy leading to endothelial injury. American journal of health-system pharmacy: AJHP: official journal of the American Society of Health-System Pharmacists.

[5] Ellulu MS, Samouda H (2022). Clinical and biological risk factors associated with inflammation in patients with type 2 diabetes mellitus. BMC endocrine disorders.

[6] International Diabetes Federation (2025). IDF Diabetes Atlas. Brussels, Belgium.

[7] Darwitz BP, Genito CJ, Thurlow LR (2024). Triple threat: how diabetes results in worsened bacterial infections. Infection and immunity.

[8] Chávez-Reyes J, Escárcega-González CE, Chavira-Suárez E, León-Buitimea A, Vázquez-León P, Morones-Ramírez JR (2021). Susceptibility for Some Infectious Diseases in Patients with Diabetes: The Key Role of Glycemia. Frontiers in public health.

[9] Giri B, Dey S, Das T, Sarkar M, Banerjee J, Dash SK (2018). Chronic hyperglycemia mediated physiological alteration and metabolic distortion leads to organ dysfunction, infection, cancer progression and other pathophysiological consequences: An update on glucose toxicity. Biomedicine & pharmacotherapy = Biomedecine & pharmacotherapie.

[10] Thiem K, Keating ST, Netea MG, Riksen NP, Tack CJ, van Diepen J (2021). Hyperglycemic Memory of Innate Immune Cells Promotes In Vitro Proinflammatory Responses of Human Monocytes and Murine Macrophages. Journal of immunology (Baltimore, Md: 1950).

[11] Gonzalez LL, Garrie K, Turner MD (2018). Type 2 diabetes - An autoinflammatory disease driven by metabolic stress. Biochimica et biophysica acta Molecular basis of disease.

[12] Wang Z, Ren J, Wang G, Liu Q, Guo K, Li J (2017). Association Between Diabetes Mellitus and Outcomes of Patients with Sepsis: A Meta-Analysis. Medical science monitor: international medical journal of experimental and clinical research.

[13] Jiang L, Cheng M (2022). Impact of diabetes mellitus on outcomes of patients with sepsis: an updated systematic review and meta-analysis. Diabetology & metabolic syndrome.

[14] Wang H, Guo Z, Zheng Y, Yu C, Hou H, Chen B (2021). No Casual Relationship Between T2DM and the Risk of Infectious Diseases: A Two-Sample Mendelian Randomization Study. Frontiers in genetics.

[15] Al Zoubi S, Chen J, Murphy C, Martin L, Chiazza F, Collotta D (2018). Linagliptin Attenuates the Cardiac Dysfunction Associated with Experimental Sepsis in Mice with Pre-existing Type 2 Diabetes by Inhibiting NF-κB. Frontiers in immunology.

[16] Frydrych LM, Bian G, Fattahi F, Morris SB, O'Rourke RW, Lumeng CN (2019). GM-CSF administration improves defects in innate immunity and sepsis survival in obese diabetic mice. Journal of Immunology.

[17] Jacob A, Steinberg ML, Yang J, Dong W, Ji Y, Wang P (2008). Sepsis-induced inflammation is exacerbated in an animal model of type 2 diabetes. International journal of clinical and experimental medicine.

[18] Jin Z, Li MY, Tang L, Zou Y, Chen K (2022). Protective effect of Ulinastatin on acute lung injury in diabetic sepsis rats. International immunopharmacology.

[19] Balintescu A, Lind M, Franko MA, Oldner A, Cronhjort M, Svensson AM (2022). Glycemic Control and Risk of Sepsis and Subsequent Mortality in Type 2 Diabetes. Diabetes care.

[20] Bitker L, Cutuli SL, Cioccari L, Osawa EA, Toh L, Luethi N (2019). Sepsis uncouples serum C-peptide and insulin levels in critically ill patients with type 2 diabetes mellitus. Critical care and resuscitation: journal of the Australasian Academy of Critical Care Medicine.

[21] Bloch O, Perl SH, Lazarovitch T, Zelnik-Yovel D, Love I, Mendel-Cohen L (2021). Hyper-Activation of Endogenous GLP-1 System to Gram-negative Sepsis Is Associated with Early Innate Immune Response and Modulated by Diabetes. Shock (Augusta, Ga).

[22] Gornik I, Gornik O, Gasparović V (2007). HbA1c is outcome predictor in diabetic patients with sepsis. Diabetes research and clinical practice.

[23] Gornik I, Vujaklija A, Lukić E, Madzarac G, Gasparović V (2010). Hyperglycemia in sepsis is a risk factor for development of type II diabetes. Journal of critical care.

[24] Jia Y, Zhao Y, Li C, Shao R (2016). The Expression of Programmed Death-1 on CD4+ and CD8+ T Lymphocytes in Patients with Type 2 Diabetes and Severe Sepsis. PloS one.

[25] Perl SH, Bloch O, Zelnic-Yuval D, Love I, Mendel-Cohen L, Flor H (2018). Sepsis-induced activation of endogenous GLP-1 system is enhanced in type 2 diabetes. Diabetes/metabolism research and reviews.

[26] Thockchom N, Bairwa M, Kant R, Kumar B, Bahurupi Y, Goyal B (2023). Prognostic Significance of Diastolic Dysfunction in Type 2 Diabetes Mellitus Patients with Sepsis and Septic Shock: Insights from a Longitudinal Tertiary Care Study. Cureus.

[27] Yagmur E, Buergerhausen D, Koek GH, Weiskirchen R, Trautwein C, Koch A (2019). Elevated CTRP1 Plasma Concentration Is Associated with Sepsis and Pre-Existing Type 2 Diabetes Mellitus in Critically Ill Patients. Journal of clinical medicine.

[28] Chang CW, Kok VC, Tseng TC, Horng JT, Liu CE (2012). Diabetic patients with severe sepsis admitted to intensive care unit do not fare worse than non-diabetic patients: a nationwide population-based cohort study. PloS one.

[29] D'Almeida SS, Moodley RM, Lameko V, Brown R (2021). Prevalence of Sepsis Continuum in Patients with Type 2 Diabetes Mellitus at Tupua Tamasese Meaole Hospital in Samoa. Cureus.

[30] de Miguel-Yanes JM, Méndez-Bailón M, Jiménez-García R, Hernández-Barrera V, Pérez-Farinós N, López-de-Andrés A (2015). Trends in sepsis incidence and outcomes among people with or without type 2 diabetes mellitus in Spain (2008-2012). Diabetes research and clinical practice.

[31] Feng S, Zhang J (2023). Clinical and Microbiological Characteristics of Type 2 Diabetes Mellitus Accompanied with Sepsis. Clinical laboratory.

[32] Gómez H, Del Rio-Pertuz G, Priyanka P, Manrique-Caballero CL, Chang CH, Wang S (2022). Association of Metformin Use During Hospitalization and Mortality in Critically Ill Adults with Type 2 Diabetes Mellitus and Sepsis. Critical care medicine.

[33] Hsieh MS, Hu SY, How CK, Seak CJ, Hsieh VC, Lin JW (2019). Hospital outcomes and cumulative burden from complications in type 2 diabetic sepsis patients: a cohort study using administrative and hospital-based databases. Therapeutic advances in endocrinology and metabolism.

[34] Hu WS, Lin CL (2024). Sodium-glucose cotransporter-2 inhibitor in risk of sepsis/septic shock among patients with type 2 diabetes mellitus-a retrospective analysis of nationwide medical claims data. Naunyn-Schmiedeberg's archives of pharmacology.

[35] Li H, Zu Y, Wang Q, Zi T, Qin X, Zhao Y (2024). Risk factor analysis and nomogram development for predicting 28-day mortality in elderly ICU patients with sepsis and type 2 diabetes mellitus. European Journal of Inflammation.

[36] Li Y, He J, Shao YM, Chen L, Li M, Tang D (2022). Study on the association between the polymorphism of MCP-1 rs1024611 and the genetic susceptibility of type 2 diabetes with sepsis. Medicine.

[37] Oh TK, Song IA (2020). Association between prior metformin therapy and sepsis in diabetes patients: a nationwide sample cohort study. Journal of anesthesia.

[38] Petrovici CG, Leca D, Teodor A, Dorneanu O, Juganariu G, Dorobăţ C (2013). Bacterial meningitis during sepsis in diabetic patient. Revista medico-chirurgicala a Societatii de Medici si Naturalisti din Iasi.

[39] Sathananthan M, Sathananthan A, Jeganathan N (2020). Characteristics and Outcomes of Patients with and Without Type 2 Diabetes Mellitus and Pulmonary Sepsis. Journal of intensive care medicine.

[40] Shih CJ, Wu YL, Chao PW, Kuo SC, Yang CY, Li SY (2015). Association between Use of Oral Anti-Diabetic Drugs and the Risk of Sepsis: A Nested Case-Control Study. Scientific reports.

[41] Sun M, Tao Y, Chen WM, Wu SY, Zhang J (2023). Optimal statin use for prevention of sepsis in type 2 diabetes mellitus. Diabetology & metabolic syndrome.

[42] Xin Q, Xie T, Chen R, Wang H, Zhang X, Wang S (2022). Predictive nomogram model for major adverse kidney events within 30 days in sepsis patients with type 2 diabetes mellitus. Frontiers in endocrinology.

[43] Yang Q, Zheng J, Chen W, Chen X, Wen D, Chen W (2021). Association Between Preadmission Metformin Use and Outcomes in Intensive Care Unit Patients with Sepsis and Type 2 Diabetes: A Cohort Study. Frontiers in medicine.

[44] Yang Y, Salam ZH, Ong BC, Yang KS (2011). Respiratory dysfunction in patients with sepsis: protective effect of diabetes mellitus. American journal of critical care: an official publication, American Association of Critical-Care Nurses.

[45] Lee EH, Lee KH, Lee K-N, Park Y, Han KD, Han SH (2025). Connection between Impaired Fasting Glucose or Type 2 Diabetes Mellitus and Sepsis: A 10-Year Observational Data from the National Health Screening Cohort. Diabetes & metabolism journal.

[46] Jin B-Y, Lee S, Kim W, Park J-H, Cho H, Moon S Association of metformin administration after septic shock with short-term and long-term survival in septic shock patients with diabetes. Annals of intensive care. 2025: 68.

[47] Weng D, Shi W, Hu Y, Chen Y, Wei S, Li A (2024). Unveiling shared diagnostic biomarkers and molecular mechanisms between T2DM and sepsis: Insights from bioinformatics to experimental assays. FASEB journal: official publication of the Federation of American Societies for Experimental Biology.

[48] Guest PC (2019). Characterization of the Goto-Kakizaki (GK) Rat Model of Type 2 Diabetes. Methods in molecular biology (Clifton, NJ).

[49] Carpenter RE (2025). Metabolic and microbial crossroads: Sodium-glucose cotransporter-2 inhibitors and urinary tract infections in (Asian) diabetes care. Health Sciences Review.

[50] Lipsky BA, Tabak YP, Johannes RS, Vo L, Hyde L, Weigelt JA (2010). Skin and soft tissue infections in hospitalised patients with diabetes: culture isolates and risk factors associated with mortality, length of stay and cost. Diabetologia.

[51] Ren M, Pan J, Yu X, Chang K, Yuan X, Zhang C (2022). CTRP1 prevents high fat diet-induced obesity and improves glucose homeostasis in obese and STZ-induced diabetic mice. Journal of translational medicine.

[52] Nagar H, Piao S, Kim CS (2018). Role of Mitochondrial Oxidative Stress in Sepsis. Acute and critical care.

[53] Sahoo DK, Wong D, Patani A, Paital B, Yadav VK, Patel A (2024). Exploring the role of antioxidants in sepsis-associated oxidative stress: a comprehensive review. Frontiers in cellular and infection microbiology.

[54] Lu J, Liu J, Li A (2022). Roles of neutrophil reactive oxygen species (ROS) generation in organ function impairment in sepsis. Journal of Zhejiang University Science B.

[55] Farkas JD (2020). The complete blood count to diagnose septic shock. Journal of thoracic disease.

[56] Agnello L, Giglio RV, Bivona G, Scazzone C, Gambino CM, Iacona A (2021). The Value of a Complete Blood Count (CBC) for Sepsis Diagnosis and Prognosis. Diagnostics (Basel, Switzerland).

[57] Wang R, Lan C, Benlagha K, Camara NOS, Miller H, Kubo M (2024). The interaction of innate immune and adaptive immune system. MedComm.

[58] Chen RY, Zhu Y, Shen YY, Xu QY, Tang HY, Cui NX (2023). The role of PD-1 signaling in health and immune-related diseases. Frontiers in immunology.

[59] Berbudi A, Khairani S, Tjahjadi AI (2025). Interplay Between Insulin Resistance and Immune Dysregulation in Type 2 Diabetes Mellitus: Implications for Therapeutic Interventions. ImmunoTargets and therapy.

[60] Fu X, Liu Z, Wang Y (2023). Advances in the Study of Immunosuppressive Mechanisms in Sepsis. J Inflamm Res.

[61] Caturano A, D'Angelo M, Mormone A, Russo V, Mollica MP, Salvatore T (2023). Oxidative Stress in Type 2 Diabetes: Impacts from Pathogenesis to Lifestyle Modifications. Current issues in molecular biology.

[62] Thimmappa PY, Vasishta S, Ganesh K, Nair AS, Joshi MB (2023). Neutrophil (dys)function due to altered immuno-metabolic axis in type 2 diabetes: implications in combating infections. Human cell.

[63] Beavers WN, Skaar EP (2016). Neutrophil-generated oxidative stress and protein damage in Staphylococcus aureus. Pathogens and disease.

[64] Cao F, Yang M, Cheng Y, Zhang X, Shi L, Li N (2023). Correlation analysis of monocyte chemoattractant protein-1 and clinical characteristics and cognitive impairment in type 2 diabetes mellitus comorbid major depressive disorder. Frontiers in Aging Neuroscience.

[65] Chen Z, Li C, Yu J (2023). Monocyte chemoattractant protein-1 as a potential marker for patients with sepsis: a systematic review and meta-analysis. Frontiers in Medicine.

[66] He J, Chen Y, Lin Y, Zhang W, Cai Y, Chen F (2017). Association study of MCP-1 promoter polymorphisms with the susceptibility and progression of sepsis. PLOS ONE.

[67] Critchley JA, Carey IM, Harris T, DeWilde S, Hosking FJ, Cook DG (2018). Glycemic Control and Risk of Infections Among People with Type 1 or Type 2 Diabetes in a Large Primary Care Cohort Study. Diabetes care.

[68] Shodja MM, Knutsen R, Cao J, Oda K, Beeson LE, Fraser GE (2017). Effects of glycosylated hemoglobin levels on neutrophilic phagocytic functions. Jacobs journal of diabetes and endocrinology.

[69] Abdelhafiz AH, Peters S, Sinclair AJ (2021). Low glycaemic state increases risk of frailty and functional decline in older people with type 2 diabetes mellitus - Evidence from a systematic review. Diabetes research and clinical practice.

[70] Cao M, Wang G, Xie J (2023). Immune dysregulation in sepsis: experiences, lessons and perspectives. Cell death discovery.

[71] Kuperman EF, Showalter JW, Lehman EB, Leib AE, Kraschnewski JL (2013). The impact of obesity on sepsis mortality: a retrospective review. BMC infectious diseases.

[72] Liu Y, Wang S, Wang Z, Yu J, Wang J, Buse JB (2024). Recent Progress in Glucose-Responsive Insulin. Diabetes.

[73] ElSayed NA, Aleppo G, Aroda VR, Bannuru RR, Brown FM, Bruemmer D (2023). 9. Pharmacologic Approaches to Glycemic Treatment: Standards of Care in Diabetes-2023. Diabetes care.

[74] Bailey CJ (2024). Metformin: Therapeutic profile in the treatment of type 2 diabetes. Diabetes, obesity & metabolism.

[75] Wang Z, Wang M, Lin M, Wei P (2024). The immunomodulatory effects of metformin in LPS-induced macrophages: an in vitro study. Inflammation research: official journal of the European Histamine Research Society [et al].

[76] Escobar DA, Botero-Quintero AM, Kautza BC, Luciano J, Loughran P, Darwiche S (2015). Adenosine monophosphate-activated protein kinase activation protects against sepsis-induced organ injury and inflammation. The Journal of surgical research.

[77] Kuai Z, Chao X, He Y, Ren W (2023). Metformin attenuates inflammation and boosts autophagy in the liver and intestine of chronologically aged rats. Experimental Gerontology.

[78] Park DW, Jiang S, Tadie J-M, Stigler WS, Gao Y, Deshane J (2013). Activation of AMPK Enhances Neutrophil Chemotaxis and Bacterial Killing. Molecular Medicine.

[79] Rossaint J, Meersch M, Thomas K, Mersmann S, Lehmann M, Skupski J Remote ischemic preconditioning causes transient cell cycle arrest and renal protection by a NF-<b>κ</b>B-dependent Sema5B pathway. 2022.

[80] Xu B, Li S, Kang B, Zhou J (2022). The current role of sodium-glucose cotransporter 2 inhibitors in type 2 diabetes mellitus management. Cardiovascular diabetology.

[81] Anush MM, Ashok VK, Sarma RI, Pillai SK (2019). Role of C-reactive Protein as an Indicator for Determining the Outcome of Sepsis. Indian journal of critical care medicine: peer-reviewed, official publication of Indian Society of Critical Care Medicine.

[82] Iannantuoni F, A MdM, Diaz-Morales N, Falcon R, Bañuls C, Abad-Jimenez Z (2019). The SGLT2 Inhibitor Empagliflozin Ameliorates the Inflammatory Profile in Type 2 Diabetic Patients and Promotes an Antioxidant Response in Leukocytes. Journal of clinical medicine.

[83] Maayah ZH, Ferdaoussi M, Takahara S, Soni S, Dyck JRB (2021). Empagliflozin suppresses inflammation and protects against acute septic renal injury. Inflammopharmacology.

[84] Chow E, Clement S, Garg R (2023). Euglycemic diabetic ketoacidosis in the era of SGLT-2 inhibitors. BMJ Open Diabetes Research & Care.

[85] Mangione CM, Barry MJ, Nicholson WK, Cabana M, Chelmow D, Coker TR (2022). Statin Use for the Primary Prevention of Cardiovascular Disease in Adults: US Preventive Services Task Force Recommendation Statement. Jama.

[86] Makhlouf HA, Hassan AK, Almosilhy NA, Osman ASA, Ramadan S, Abouelmagd ME (2025). Exploring the association between statins use or HMG-CoA reductase inhibition and migraine: a systematic review and meta-analysis. The Journal of Headache and Pain.

[87] Rachoin JS, Cerceo E, Dellinger RP (2013). A new role for statins in sepsis. Critical care (London, England).

[88] Chen M, Ji M, Si X (2018). The effects of statin therapy on mortality in patients with sepsis: A meta-analysis of randomized trials. Medicine.

[89] Rajpathak SN, Kumbhani DJ, Crandall J, Barzilai N, Alderman M, Ridker PM (2009). Statin therapy and risk of developing type 2 diabetes: a meta-analysis. Diabetes care.

[90] Saini K, Sharma S, Khan Y (2023). DPP-4 inhibitors for treating T2DM - hype or hope? an analysis based on the current literature. Frontiers in molecular biosciences.

[91] Pratley RE, Gilbert M (2008). Targeting Incretins in Type 2 Diabetes: Role of GLP-1 Receptor Agonists and DPP-4 Inhibitors. The review of diabetic studies: RDS.

[92] Zhang D, Song J, Zhan J, Wang Y, Deng J, Deng Y (2024). The impact of ulinastatin on lymphocyte apoptosis and autophagy in sepsis patients. Scientific reports.

[93] Wang H, Liu B, Tang Y, Chang P, Yao L, Huang B (2019). Improvement of Sepsis Prognosis by Ulinastatin: A Systematic Review and Meta-Analysis of Randomized Controlled Trials. Frontiers in pharmacology.

